# ICEKp2: description of an integrative and conjugative element in *Klebsiella pneumoniae*, co-occurring and interacting with ICEKp1

**DOI:** 10.1038/s41598-019-50456-x

**Published:** 2019-09-25

**Authors:** Robeena Farzand, Kumar Rajakumar, Roxana Zamudio, Marco R. Oggioni, Michael R Barer, Helen M. O’Hare

**Affiliations:** 1 Leicester Microbial Sciences and Infectious Diseases Network, Department of Respiratory Sciences, University of Leicester, Leicester, UK; 2 Kohat University of Science and Technology, Department of Microbiology, Kohat, Pakistan; 3 Department of Genetics and Genome Biology, University of Leicester, Leicester, UK; 4 Department of Molecular and Cell Biology, University of Leicester, Leicester, UK

**Keywords:** Bacteriology, Pathogens, Microbial genetics

## Abstract

*Klebsiella pneumoniae* is a human pathogen, prominent in antimicrobial-resistant and nosocomial infection. The integrative and conjugative element ICEKp1 is present in a third of clinical isolates and more prevalent in invasive disease; it provides genetic diversity and enables the spread of virulence-associated genes. We report a second integrative conjugative element that can co-occur with ICEKp1 in *K. pneumoniae*. This element, ICEKp2, is similar to the *Pseudomonas aeruginosa* pathogenicity island PAPI. We identified ICEKp2 in *K. pneumoniae* sequence types ST11, ST258 and ST512, which are associated with carbapenem-resistant outbreaks in China and the US, including isolates with and without ICEKp1. ICEKp2 was competent for excision, but self-mobilisation to recipient *Escherichia coli* was not detected. In an isolate with both elements, ICEKp2 positively influenced the efficiency of plasmid mobilisation driven by ICEKp1. We propose a putative mechanism, in which a Mob2 ATPase of ICEKp2 may contribute to the ICEKp1 conjugation machinery. Supporting this mechanism, *mob2*, but not a variant with mutations in the ATPase motif, restored transfer efficiency to an ICEKp2 knockout. This is the first demonstration of the interaction between integrative and conjugative genetic elements in a single Gram-negative bacterium with implications for understanding evolution by horizontal gene transfer.

## Introduction

*Klebsiella pneumoniae* is an important pathogen contributing to nosocomial and antimicrobial resistant infections. The strain studied here, *K. pneumoniae* HS11286 (CP003200.1), is a carbapenemase (KPC-2) producing, multidrug-resistant (MDR) clinical isolate^1,2^ belonging to sequence type ST11, which is a dominant carbapenem resistant clone in China^3^ and is closely related to a worldwide dominant carbapenem resistant *K. pneumoniae* clone ST258^4,5^. The acquisition and spread of antimicrobial resistance-associated and virulence-associated genes is facilitated by certain types of mobile genetic element (MGE) including plasmids, insertion sequences, transposons, integrons and associated gene cassettes, prophages, self-transmissible integrative and conjugative elements (ICEs) and genomic islands^6,7^.

MGEs are capable of horizontal gene transfer and hence are potential agents for the spread of virulence or amtimicrobial resistance genes. *K. pneumoniae* carries many MGE, two of which have been linked to virulence: ICEKp1, a 62 kb chromosomal island integrated at *tRNAasn*, and plasmid pLVPK. These elements are more prevalent in invasive disease isolates, and pLVPK influences capsule type^1,8^. Intriguingly, sequencing of our strain of interest, HS11286, revealed a second putative ICE in addition to ICEKp1^1,9,10^. The co-occurrence of multiple MGEs brings the potential for functional interactions that could promote mobilisation. The integrases of one pathogenicity island can increase excision of another^11,12^, and an ICE has been observed to mobilise an Integrative and Mobilisable Element (IME)^13^. To our knowledge, functional interaction between two ICE in the same genome has not previously been demonstrated. The second putative ICE, ICEKp2, is a 56 kb chromosomal island integrated at *tRNAphe*. Here we identify a complete set of genes for mobilisation on the basis of homology of the integration module and conjugative machinery with those of the *Pseudomonas aeruginosa* pathogenicity island family (PAPI)^14,15^.

Many PAPI-like elements, including PAPI-1, PAGI-1, PAGI-5, pKCL102, and PFGI-1 have been identified in various species of *Pseudomonas*, where they are linked to virulence^10,16–19^. A PAPI-like element was also reported in *E. coli* BEN374 (ICEEc2) and shown to be capable of excision, though mobilisation remains incompletely characterised^20^. For comparison, excision and mobilisation have been demonstrated for ICEKp1 of *K. pneumoniae* NTUH-K2044^21^.

To determine the evolutionary and functional relationship between the newly annotated PAPI-like ICEKp2 and the more widespread ICEKp1, we undertook annotation and functional characterisation of ICEKp2 to determine its potential for self-mobilisation, DNA mobilisation in *trans*, and influence on DNA mobilisation by ICEKp1.

## Results

### The putative integrative conjugative element is a member of the PAPI family

**Figure 1:**
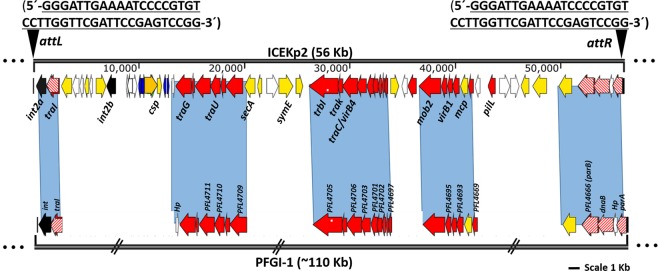
Comparison of the unusual integrative conjugative element ICEKp2 of *K. pneumoniae* HS11286 with PFGI1 of *Pseudomonas fluorescens* Pf-5 classifed ICEKp2 as a member of the PAPI family. ICEKp2 contains sufficient predicted ORFS for mobilisation: recombination, DNA segregation, and conjugation (T4SS and relaxase), plus 28 cargo genes. Homology and synteny of the mobilisation genes place it in the PAPI family. Predicted open reading frames (ORFs) are shaded according to their putative functions (ORFs are listed in table S1). Black triangles on ICEKp2 boundaries represent the direct repeat sequences for chromosomal integration to generate *attL* and *attR* sites. Further information on the annotation of ORFs is provided in Table S1. Blue shading represents the regions of synteny of DNA mobilisation and integration modules between ICEKp2 and PFGI-1 and PAPI of other *Pseudomonas* spp.^18,66^.

We performed functional annotation of the putative ICEKp2 to confirm its identity as an integrative conjugative element by annotation of the sequence motifs and ORFs typically involved in excision and transfer. High homology (40–85% amino acid identity) and synteny of 22 DNA mobilisation/conjugation genes (Fig. 1) place the element in the PAPI family. We identified the expected modules for conjugation in ICEKp2: integrase for recombination, a full set of ORFs for DNA mobilisation and conjugation via a type 4 secretion system (by comparison with transfer-efficient PAPI of *Pseudomonas* species)^18,22,23^. Uniquely among conjugative genetic elements, ICEKp2 contains two integrase genes, *int2a* and *int2b*, whereas ICEKp1 and other PAPI have a single integrase. Annotated ORFs are listed in Table S1.

**Figure 2:**
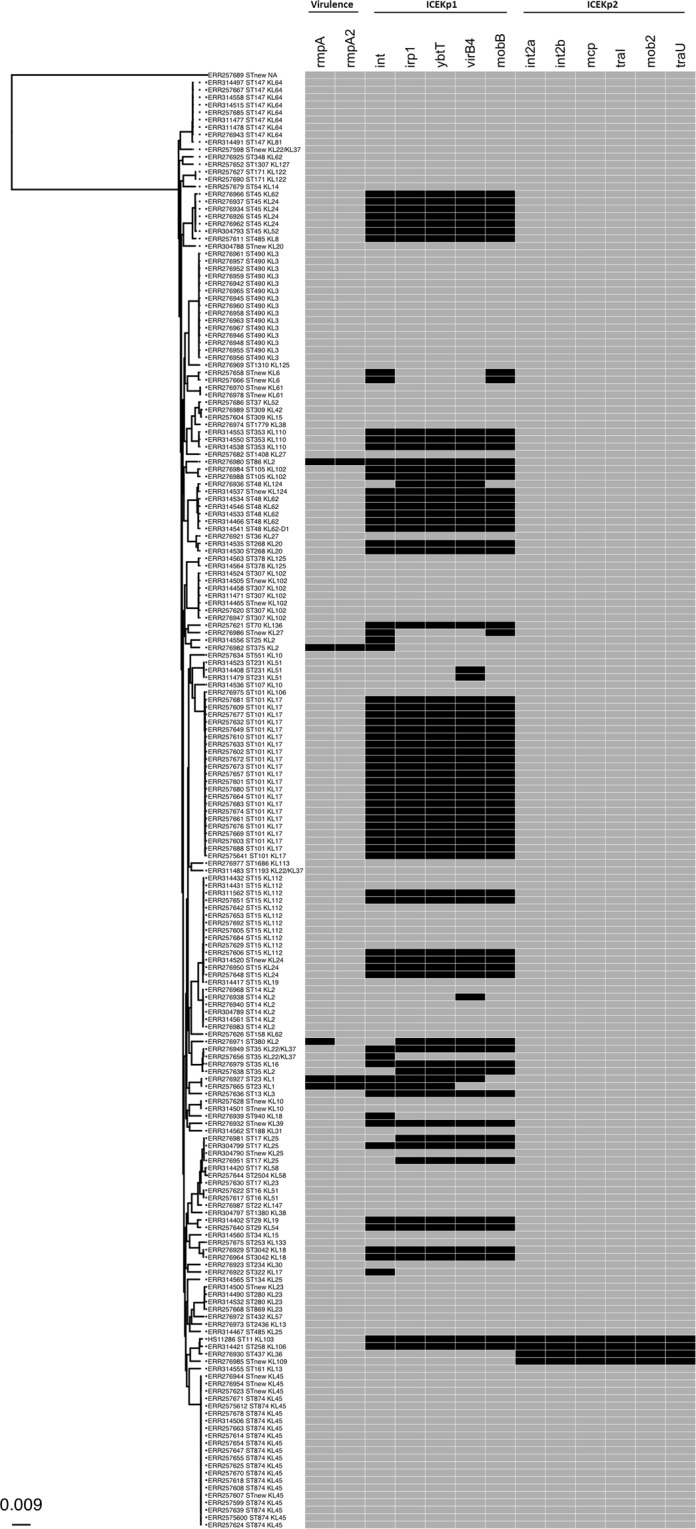
ICEKp2 was recognised in three UK isolates sequenced in two previous studies and appears restricted to closely related sequence types. The core genome phylogenetic tree was generated for 196 *K. pneumoniae* isolates (from studies described in^24,25^, as described in Table S2). The sequence type (ST) and capsule synthesis loci (KL) of all isolates is shown including study strain HS11286. The occurrence and co-occurrence of ICEKp1 and ICEKp2 (marker genes) are shown in the heat map to the right (black = present, grey = absent). The virulence associated genes *rmpA* and *rmpA2* were also included in the analysis to determine their correlation with the presence/absence of ICEKp1 and ICEKp2. The scale bar shown at the bottom left represents the average number of substitution per site. The scale bar shown at the bottom left represents the average number of substitutions per site.

**Figure 3:**
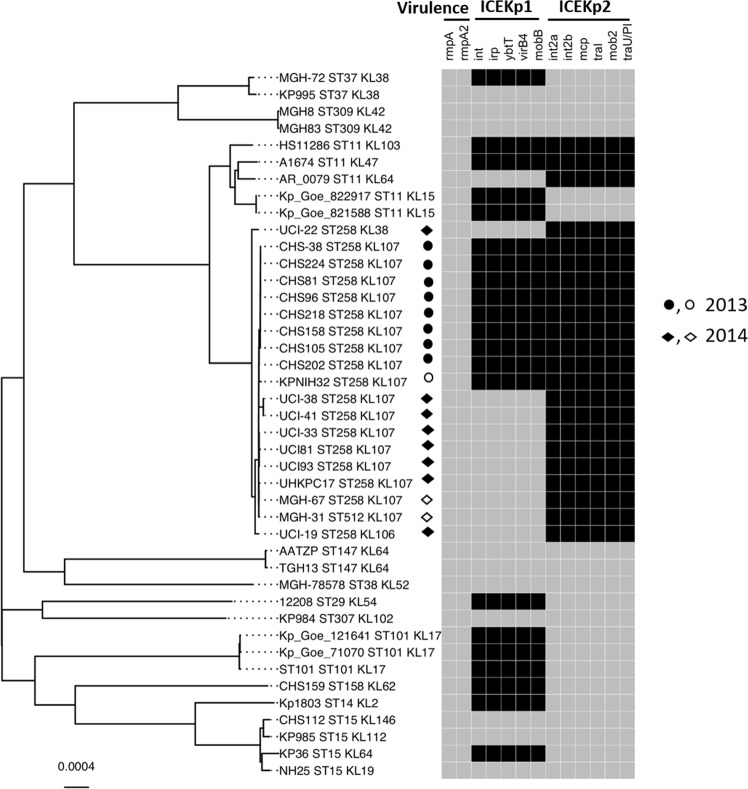
Whole genome database searches revealed that ICEKp2 occurred in two outbreaks of carbapenem resistant *K. pneumoniae* in the US, in isolates with and without ICEKp1. The core genome tree shows the sequence type (ST) and capsule synthesis loci (KL) of all isolates and the study strain HS11286. The occurrence and co-occurrence of ICEKp1 and ICEKp2 (marker genes) are shown in the heat map to the right (black = present, grey = absent). The virulence associated genes *rmpA* and *rmpA2* were also included in analysis to determine their co-relation with the presence/absence of ICEKp1 and ICEKp2. For US isolates, the shape indicates the year of isolation: circles for 2013 and diamonds for 2014, and shading indicates that the strain was part of an outbreak (open shapes were not outbreak associated). The scale bar shown at the bottom left represents the average number of substitutions per site.

To determine the prevalence of ICEKp2 in *K. pneumoniae* clinical isolates, we analysed whole-genome sequences of 196 UK isolates from two previous studies^24,25^ (Table S2) and identified ICEKp2 in three strains that are closely related to our study strain: sequence types ST258, ST437 and a new ST (Fig. 2). One of those also carried ICEKp1. Additionally, we analysed 40 local clinical isolates for the presence of ICEKp2 and ICEKp1 by PCR and found this element in two, neither of which carried ICEKp1 (Fig. S1). To further determine the presence of ICEKp2 in different sequence types, and to investigate whether the presence of ICEKp2 correlates with ICEKp1, we interrogated 1000 *K. pneumoniae* complete genome sequences from NCBI (selected by completeness). We identified ICEKp2 in 300 genomes of closely related sequence types (81.6% were of ST258) (Table S3). The majority were sequenced from two US outbreaks of carbapenem resistant infections in 2013 and 2014 (details are provided in Table S4). ICEKp2 was restricted to closely related sequence types, suggesting stable inheritance of ICEKp2. ICEKp1 was present in 500 of the 1000 genomes and its presence was not correlated with ICEKp2 (Table 1). Core genome phylogeny of the representative strains is shown in Fig. 3 (Table S5).

**Table 1:** Occurrence and co-occurrence of ICEKp1 and ICEKp2 in *K. pneumoniae* genomes investigated in this study.

Occurrence	Whole genome sequences NCBI (n = 1000)	Whole genome sequences UK-isolates (n = 196)	Leicester isolates (n = 40)
ICEKp1	500	60	11
ICEKp2	300	3	2
Co-occurrence	150	1	none

Amongst our analysed strains, ICEKp2 did not co-occur with the plasmid carrying mucoid factor encoding genes *rmpA* and *rmpA2* that are associated with hyper-expression of K1 or K2 capsule types and increased virulence^8,26^. By contrast, *K. pneumoniae* possessing this plasmid always carried a partial or complete ICEKp1 (Fig. 2). Furthermore, the frequency of ICEKp1 was higher for some sequence types than others, for example, 100% in ST45, ST353, and ST105 compared to 36% in ST15 (5/14 isolates, Fig. 2). This could be suggestive of a more ancient origin and/or greater mobility of ICEKp1 compared to ICEKp2.

**Figure 4:**
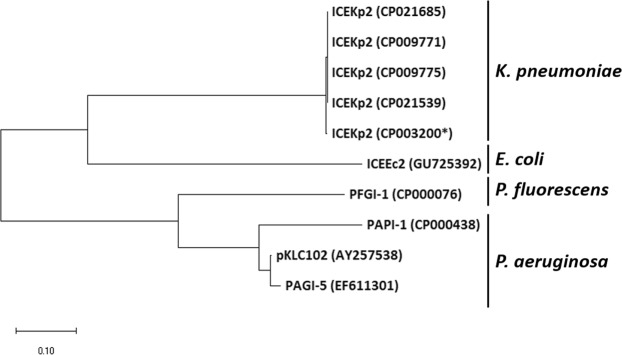
Phylogenetic analysis of integrases of ICEs from *K. pneumoniae*, *P. aeruginosa, P. fluorescens* and *E. coli* grouped ICEKp2 separately from those in the other organisms. PAPI-1^66^, pKLC102^23^, PAGI-5^22^ are PAPI members of *P. aeruginosa*, whereas PFGI-1 is PAPI reported in plant commensal *P. fluorescens*^18^. ICEEc2 is the first PAPI member reported in *E. coli*^20^. ICEKp2 (CP003200*) is the ICE of *K. pneumoniae* strain HS11286 studied in this work, and the other PAPI of *K. pneumoniae* were identified from whole genome sequences and are named according to the strain. The bar show changes observed between two sequences; 0.1 means 10% amino acid changes.

Unlike ICEKp1, which is variable in terms of cargo genes^27^, we found that ICEKp2 is highly conserved: all 28 cargo ORFs were present and highly conserved (>90% amino acid identity) in all 304 ICEKp2 elements detected. Accordingly, ICEKp2 elements group together phylogenetically, and are distinct from PAPI of *P. aeruginosa* and *E. coli* (Fig. 4). The closest conjugative genetic element was ICEEc2 (PAPI of *E. coli*), based on nucleotide identity within the core genes^20^. Like ICEEc2, ICEKp2 is integrated into *tRNAphe*, whereas PAPI of *Pseudomonas* species are found in *tRNAlys*. None of the cargo genes are conserved between ICEKp2 and ICEEc2, indeed many of the ICEKp2 cargo genes are of unknown function (Table S1). Seven of the cargo genes may influence the ability of *K. pneumoniae* to adapt to environmental changes during infection as they encode a predicted cold shock protein, toxin-antitoxin system, a chemotaxis signalling protein (methyl-accepting chemotaxis protein), and three transcriptional regulators (annotated by homology)^28–33^.

### ICEKp2 is capable of integrase-mediated excision but not efficient DNA transfer

**Figure 5:**
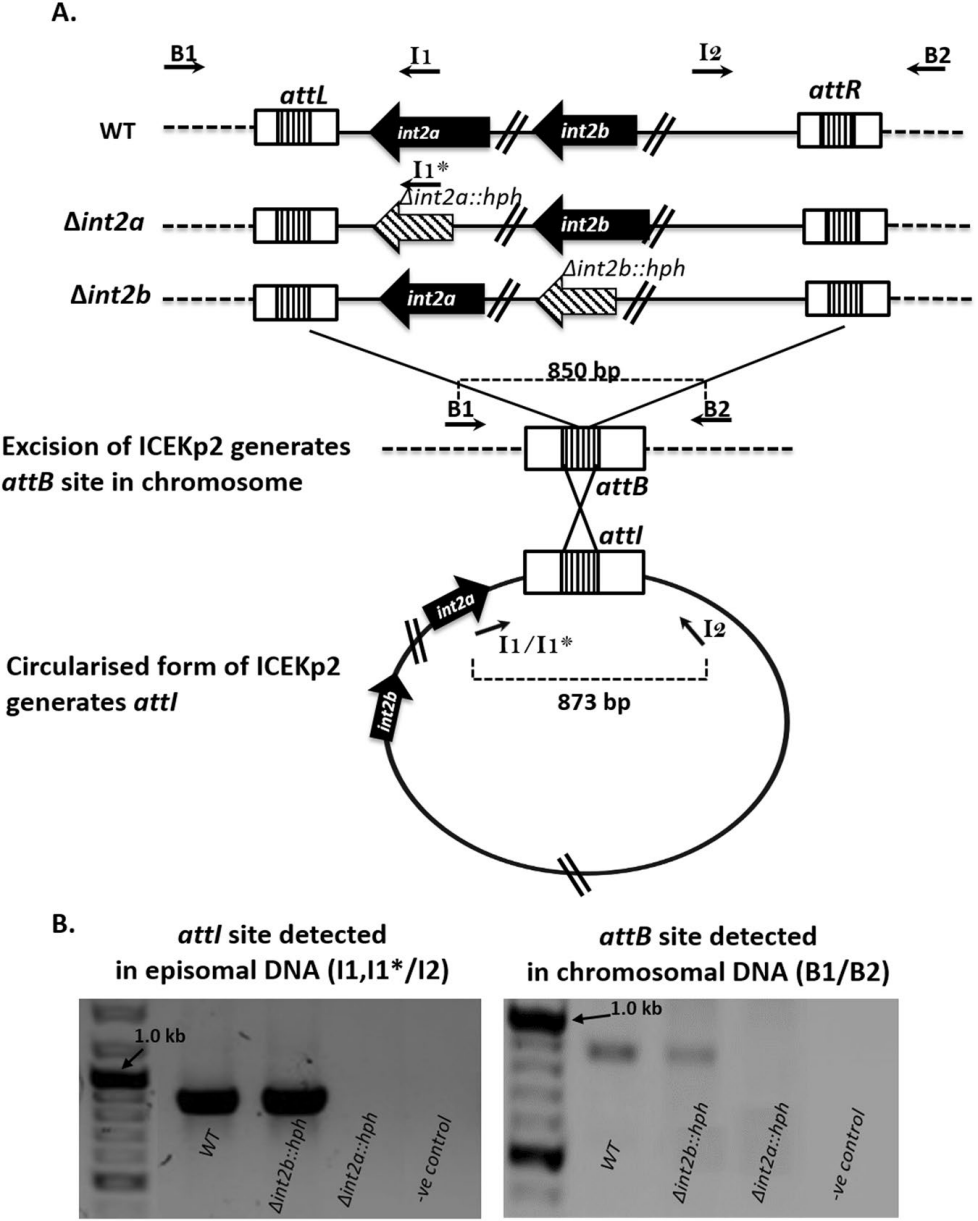
PCR detection of site-specific excision and extrachromosomal circularisation of ICEKp2. (**A**) Panel A shows ICEKp2 in the chromosome of the study strain wild type (WT) and the *int2a* and *int2b* mutants that were constructed, as well as the expected excised form of ICEKp2 and the expected empty *attB* site on the chromosome following excision. Primers were designed to discriminate between the different *att* sites before and after excision: primers B1 + B2 amplify the empty *attB* site (scar) following excision, and primers I1 and I2 amplify the *attI* site in excised ICEKp2. A variant primer, I1* was designed for use with *int2a* deletion strain to prime in the *hph* gene. (**B**) Amplification of the excised form of *attI* from episomal DNA demonstrated excision of ICEKp2 from *K. pneumoniae* with ICEKp2 (WT = wild type) and from *K. pneumoniae* carrying the *int2b* mutant of ICEKp2, but not from *int2a* mutant strain (left panel). This result was confirmed independently by amplification of the scar *attB* site from the chromosomal DNA of WT and *int2b* mutant but not *int2a* mutant (right panel). We verified that both I1 and I1* primers annealed efficiently on their respective sequences by a control amplification of the *attL* site (Fig. S2).

Our sequence annotation of ICEKp2 identified the necessary components for excision, namely direct repeat sequences and integrases (Fig. 1, and Table S1). To determine the functionality of these elements, we designed PCR assays for specific detection of putative circularised excised ICEKp2 and the post-excision *attB* site in the chromosome (Fig. 5). Both types of assay indicated excision of ICEKp2 in the study strain (wild type). To determine which of the two integrases are functional, we constructed mutant strains lacking *int2a* or *int2b* and used the same assays to probe for excision. Excision was detected for the strain lacking *int2b* but not for the strain lacking *int2a* (Fig. 5), despite the verified functionality of the primer I1* with the relevant strain (Fig. S2), thus Int2a was identified as the likely integrase responsible for the excision of ICEKp2.

Once an element is excised, additional elements are required for mobilisation to another bacterial cell, namely a type IV secretion system (T4SS), relaxase and origin of transfer. A complete putative T4SS and relaxase (ORF2) were identified in ICEKp2 (Fig. 1 and Table S1), whereas the origin of transfer is typically difficult to predict in ICE elements. To detect putative transfer of ICEKp2 to *E. coli* by self-mobilisation, we inserted a hygromycin resistance marker into cargo gene ORF8 of ICEKp2 and screened for transfer of hygromycin resistance from the resulting *K. pneumoniae* strain to *E. coli*. No hygromycin resistant colonies were obtained from 10^7^ *E. coli*, thus self-transfer was inefficient or absent.

We next deleted ICEKp1 to investigate whether ICEKp2 could mobilise marker plasmids. We used a panel of marker plasmids that span the whole ICEKp2 element to test all possible origins of transfer (marker plasmids are shown in Fig. S3 and the 35 repeat sequences that could be the candidate origin of transfer are shown in Table S6; a plasmid with the ICEKp1 origin of transfer was also tested as a control). No ICEKp2-dependent mobilisation was detected, whereas a parallel experiment using the study strain (wild type, which contains ICEKp1) generated 100 colonies by mobilisation of the marker plasmid carrying the ICEKp1 origin of transfer.

### ICEKp1-driven mobilisation was enhanced by the presence of ICEKp2, and this required functional *mob2*

**Figure 6:**
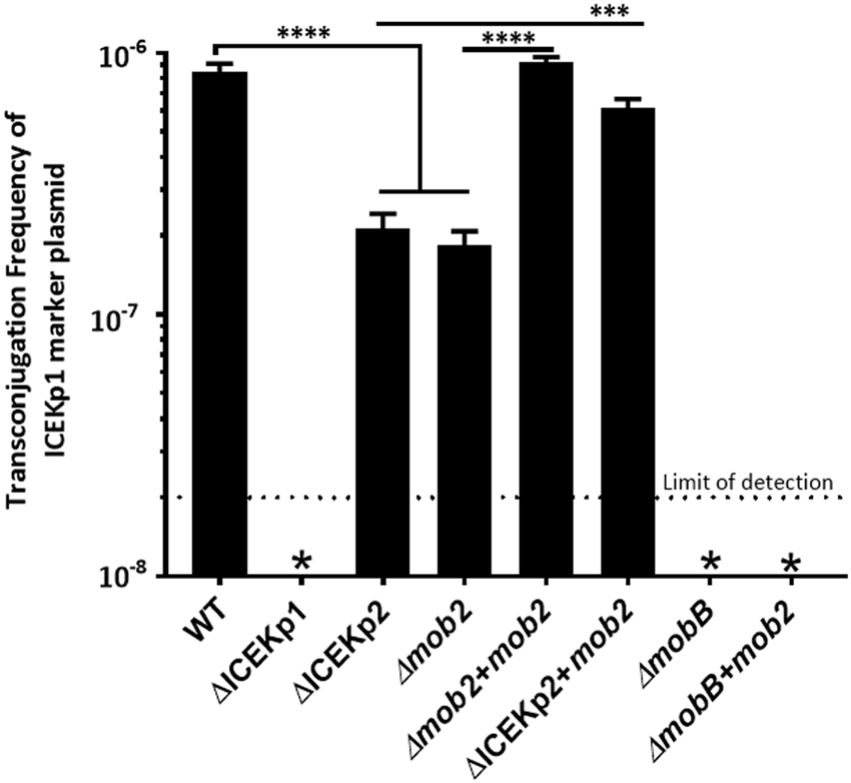
*mob2* of ICEKp2 increased plasmid mobilisation by ICEKp1. The functional interaction between ICEKp2 and ICEKp1 was investigated by measurement of conjugal transfer of ICEKp1 marker plasmid to *E. coli* from *K. pneumoniae* containing ICEKp1 and variants of ICEKp2. Deletion of ICEKp2 or *mob2* led to a 6-fold reduction in conjugal transfer of the marker plasmid, which was restored by reintroduction of *mob2* (+*mob2*). *mob2* did not complement a *mobB* deletion in ICEKp1, demonstrating that *mobB* and *mob2* are not interchangeable. (Error bars = Standard deviation). ****P-value < 0.0001, which is representative of six independent experiments.

Co-occurrence of excision-competent ICEs, each encoding proteins involved in gene transfer, allows the potential for interaction between the different ICEs. To test this hypothesis, we deleted ICEKp2 from the study strain and found a reduction in the frequency of ICEKp1-mediated plasmid mobilisation (Fig. 6), while the survival rate of the donor strain was not changed significantly (Table S8).

A possible mechanism for interaction between the different elements would include shared components of the conjugation machinery. ICEKp1 encodes a full T4SS that is responsible for DNA transfer^21^ and ICEKp2 also encodes sufficient T4SS components for possible mobilisation (Fig. 1 and Table S7). The T4SS from ICEKp1 and ICEKp2 are from different T4SS families and so the proteins would not be expected to interact with each other. However, a putative type 4 coupling protein from ICEKp2, which we designate Mob2, is from the same family (VirB/VirD4) as that in ICEKp1 (MobB). Coupling proteins are ATPases that provide energy for transfer, and a coupling protein has been reported to mediate cross-talk between two T4SS, although these were not part of MGEs^34^. Mob2 shares 48% amino acid identity with MobB and in principle it could contribute to the ICEKp1 T4SS conjugation machinery.

We found that *mob2* cannot replace the function of *mobB*, since deletion of *mobB* in the study strain carrying both ICE resulted in the loss of plasmid mobilisation despite the continued presence of *mob2* (Fig. 6). To determine whether Mob2 can influence ICEKp1-driven mobilisation, we made mutations in ICEKp2 and measured ICEKp1-driven marker plasmid transfer. Deletion of *mob2* alone reduced transfer to the same extent as deletion of the whole ICEKp2 (Fig. 6), and transfer efficiency of both the ICEKp2 deletion strain and *mob2* depletion strain was restored by the reintroduction of *mob2*. By contrast, a variant of *mob2* with mutations in the ATP binding site (Walker A and B motifs) did not increase ICEKp1-driven plasmid transfer (Fig. S4).

## Discussion

ICE are widely recognised as contributing to the spread of AMR and pathogenicity. For most MGEs, there are recognised examples of two (or more) of an individual MGE-type occurring in the same host^35,36^. As far as we know, this study represents the first description of functional interaction between two ICE. Cross-talk has been observed between *Vibrio* pathogenicity islands, mediated by cross-talk of the integrases to promote excision^12^. The evidence we present of cross-talk between ICEs is Mob2-mediated increase in the efficiency of ICEKp1-driven plasmid mobilisation.

ICEKp2 is highly related to *Pseudomonas* PAPI, which is more widespread in *Pseudomonas* than in *Enterobacteriaceae*. Our analysis showed ICEKp2 distribution was restricted to the US, UK, and Asian isolates. Unlike PAPI in *Pseudomonas*, or ICEKp1, for which different members of the family contain different cargo genes, all the ICEKp2 that we identified have the same cargo genes and there was little sequence diversity. Together with the core genome phylogenetic analysis (Fig. 2), this suggests relatively recent acquisition by *K. pneumoniae*, followed by vertical transmission. ICEKp2 was limited to closely related *K. pneumoniae* sequence types.

Despite the apparent complete set of mobilisation genes in ICEKp2, and the competence of ICEKp2 to self-excise, we did not detect independent mobilisation by ICEKp2 and we failed to identify an origin of transfer *oriT* of ICEKp2. Various mechanisms have been reported for loss of self-mobilisation of other conjugative elements, which can then be maintained by vertical transmission and selective advantage. One possible selective pressure for maintenance of ICEKp2 could be the toxin/antitoxin system in ICEKp2 (as seen for other TA systems^37^). However, our ability to delete ICEKp2 argues against strong selection by this mechanism.

One mechanism underlying loss of self-mobilisation is mutational inactivation or loss of the necessary elements for mobilisation, for example, the partial versions of ICEKp1 (Fig. 2). By a similar mechanism, a MGE can evolve to rely on genes from another MGE within the same genome in order to mobilise, or to rely on genes from the core genome. Integrative and Mobilisable Elements, for example, rely on the conjugative machinery of other elements, and the IMEs characterised to date are distinct from ICE by their lack of genes encoding conjugation apparatus^13^. An example of reliance on the core genome is certain PAPI, for which PilD encoded by the core genome processes the T4SS^38^. Expression of the mobilisation apparatus has a fitness cost and bacteria can transition between low and high frequencies of conjugation^39,40^. Being competent for self-excision, ICEKp2 might be mobilisable by another element such as ICEKp1, but there was no evidence of correlation of occurrence of ICEKp1 with ICEKp2 (Table 1).

Our sequence analysis indicated substantial rearrangements in the genetic organisation of the T4SS system in ICEKp2 compared to PAPI in *Pseudomonas* (Fig. 1), suggesting loss of functionality of the conjugation system as a potential mechanism for loss of self-transmission. Generally, the gene clusters encoding the conjugation system in the PAPI group are intact and organised into a single operon^18^, whereas the components of T4SS are divided between more than one locus in ICEKp2 (Fig. 1); this could affect the regulation of gene expression.

The capsule type influences the efficiency of horizontal gene transfer^41^, in particular mucoid capsule types, K1 and K2 have more accessory genomes, possibly indicating more efficient horizontal gene transfer^42,43^. Within our analysis, ICEKp2 did not occur in mucoid strains. By contrast, mucoid capsule types were positively associated with ICEKp1 (Fig. 2), as reported by others recently^42^.

The presence of two T4SS in a cell containing both ICEKp1 and ICEKp2 could allow functional interaction between the two systems, as has been observed in *Agrobacterium* de Paz, *et al*.^34^, although these T4SS were not part of MGEs. Our observation that ICEKp2 influenced the efficiency of DNA mobilisation by ICEKp1 could be related to interaction between the T4SS.

To identify candidate genes from ICEKp2 T4SS that might interact with ICEKp1 we analysed the annotated functions (Table S7). Genes involved in pilus assembly were considered unlikely to interact with ICEKp1 because they are highly specific for their own systems^44^. On the other hand, coupling proteins from diverse conjugation systems share common features^14^, and we hypothesised that coupling protein Mob2 might enhance ICEKp1 mediated transfer. Mob2 is part of the TraD family of coupling proteins, which mediate DNA transport to the secretion channel by interacting with both the relaxosome and the secretory channel^45,46^. In conjugative transfer, the coupling protein delivers the DNA substrate to VirB11 and this is translocated to the recipient via the VirB2 channel^47^. Cascales, *et al*.^48^. For DNA translocation, an energy hub is provided by three ATPases (VirB4, VirB11, and a coupling protein) and this is referred to as the cytoplasmic power house of conjugation^49,50^. The coupling protein senses intracellular signals from the relaxosome and processes the nucleoprotein complexes to the secretion channel utilizing its ATPase activity^51–54^. Mob2 might enhance T4SS (ICEKp1) mediated transfer by contributing energy. The recognition that MobB apparently lacks a Walker B motif in all ICEKp1, whereas ICEKp2 Mob2 appears to contain an intact Walker B motif, adds further weight to this possibility. We found that *mob2* was necessary and sufficient for the enhancement of ICEKp1-mediated conjugative transfer, and that this activity required the intact Walker B motif of *mob2*, suggesting that ATP hydrolysis may be involved.

In conclusion, we report the occurrence of a PAPI-related ICE in *K. pneumoniae* in clinical isolates from multiple continents. While currently infrequently associated with UK isolates, ICEKp2 has been associated with outbreaks in the USA. In our isolate, we demonstrated that *mob2* from ICEKp2 enhanced mobilisation of co-occurring ICEKp1, adding further to the range of interactions facilitating the dissemination of virulence and antimicrobial resistance by horizontal gene transfer.

## Methods

### Strains, media, and culture conditions

The study strain *K. pneumoniae* HS11286 was isolated at the Shanghai Jiaotong University (SJTU), China, and was kindly provided by Professor Hong-Yu OU (School of Life Sciences & Biotechnology). A further 40 clinical isolates were obtained from the Leicester Royal Infirmary (LRI) Hospital, UK (collected from June-December 2011). In each case, strains were stored at −80 °C in BHI (Brain Heart Infusion) broth with 30% glycerol. The strains were routinely grown at 37 °C using Luria-Bertani agar (LA) and broth (LB). When needed, antibiotics were used at the following concentrations: 100 µg/ml ampicillin, 50 µg/ml kanamycin, 30 µg/ml apramycin, 100 µg/ml hygromycin, 50 µg/ml streptomycin, 30 µg/ml chloramphenicol.

### Annotation and characterisation of mobile genetic elements

The functional annotation of genes predicted by ORF Finder was achieved by Rapid Annotation Subsystem Technology (RAST) server (http://rast.nmpdr.org/rast.cgi, ^55^). Annotation of genes (Tables S1) was manually confirmed using the BLAST (nucleotide and protein) servers of the National Center for Biotechnology Information (http://www.ncbi.nih.gov). A motif search was performed using the Pfam server (https://pfam.xfam.org/), and CDD server (https://www.ncbi.nlm.nih.gov/cdd/). Pairwise sequence alignment and multiple alignments of nucleotide or protein sequences were performed using EMBL-EBI services EMBOSS and MUSCLE. REPuter was used to detect repeated sequences in ICEKp2^56^.

### Whole genome analysis

The draft genomes were annotated using Prokka (version 1.11)^57^ and the pangenome analysis was done using Roary (version 1.007001)^58^ using 80% identity blastp. 3,061 core genes were identified and then gene-by-gene aligned using Muscle (version 3.8.31)^59^ and afterward concatenated using a custom python script. The maximum-likelihood core genome phylogenetic tree was inferred from the concatenated alignment core genes (2,977,141 bp) using the GTR model in RAxML (Randomized Axelerated Maximum Likelihood) (version 8.2.12) with 2849 and 3849 genes for Figs 2, 3 respectively^60^. Add here the gene numbers for Figs 2, 3. The presence of the mobile elements genes was scanned in the draft genomes using blastn by setting identity >82% and coverage >88%. The ggtree version 1.13.1 R package was used for the visualization of the phylogenetic tree with integrated mobile elements data. In silico MLST was performed using a BLAST-based tool (https://github.com/tseemann/mlst) on draft genomes. Phylogenetic analysis of integrase amino acids sequences by maximum-likelihood method used MEGA10^61^. Bootstrap values were calculated with 1000 replications.

### PCR detection of integrated and excised ICEKp2

Genomic DNA was isolated from 500 μl of dense culture using Archive DNA Cell/Tissue Kit (5Prime), and extrachromosomal DNA was extracted from a second aliquot of the same bacterial culture using GenElute Plasmid Miniprep kit (Sigma-Aldrich). The purified DNA was tested for the presence of ICEKp2 sequences by PCR reactions, using either 10 or 100 ng per 25 μl PCR reaction. To amplify either the chromosomal junction or the episomal state of ICEKp2, minor modifications were made to the primers described Lin, *et al*.^21^ Schubert, *et al*.^62^. Primers are listed in supplementary Table S9 and the PCR validation for detection of excision of wild type and mutated ICEKp2 is shown in Fig. S1. For PCR KOD Hot start DNA polymerase (Merck Millipore) was used and amplicons were analysed both by agarose gel electrophoresis and amplicon sequencing (GATC-Eurofins).

### Generation of *K. pneumoniae* mutant strains

Mutants of *K. pneumoniae* HS11286 were generated by the 3-step lambda red recombination method described earlier^63^. Briefly, the upstream and downstream regions of the targeted gene/genes were amplified and assembled on either side of a hygromycin resistance cassette (*hph*) by overlap extension PCR. The product was transformed into *K. pneumoniae* cells previously induced with arabinose for the expression of lambda Red protein encoded by pKOBEG plasmid. The in-frame deletion of targeted gene/genes and integration of *hph* cassette was then confirmed by PCR and sequencing (GATC-Eurofins).

### Construction of plasmid P-oriT-1

A marker plasmid (P-oriT-1) was constructed to measure plasmid mobilisation activity of the conjugation module of ICEKp1 and potential activity of the conjugation module of ICEKp2. The region of ICEKp1 containing the origin of transfer *oriT* (a 1760 bp region between *virB11* and *mobB*) was amplified from *K. pneumoniae* HS11286 genomic DNA using primers oriT-HindIII_F and oriT-SalI_R (Table S9) and cloned into the HindIII and SalI sites of pACYC184. The plasmid pACYC184 was chosen because it has already been used to demonstrate DNA mobilisation by ICEKp1 in other strains of *K. pneumoniae*^21,62^. Verification of transfer of the newly constructed plasmid P-oriT-1 by *K. pneumoniae* HS11286 (which contains ICEKp1) was performed by selection for transfer of chloramphenicol resistance using the filter mating protocol below (Fig. S5).

### Conjugation by filter mating

A colony of *K. pneumoniae* HS11286 (or mutant) containing the relevant marker conjugative plasmid was used to inoculate LB (5 ml). Separately, a colony of *Escherichia coli* HB101 was also used to inoculate LB (5 ml). After incubation overnight, the cultures were washed twice with PBS and then mixed in the ratio of 10:1 *K. pneumoniae* (donor) to *E. coli* (recipient) in 200 µl PBS (ratio based on colony-forming units). The mixture was placed on a 0.22 µm pore size membrane filter (Thermo Fisher) on LA and incubated overnight. Cells from the membrane were then resuspended in 1 ml PBS and plated on LA supplemented with both chloramphenicol and streptomycin, or other antibiotics as stated. The efficiency of transconjugation was calculated from the number of transconjugants per recipient. PCR was used to confirm the presence of the P-oriT-1 in transconjugants using plasmid-specific primers (Table S9) and, in parallel; transconjugants were verified as *E. coli* using primers specific to *E. coli*.

### Construction of RFTool-1 and complementation studies

To reintroduce genes to mutant strains, an expression plasmid RFTool-1 was constructed from pWSK29^64^, which has been used for protein expression in *K. pneumoniae*. The ampicillin resistance cassette of pWSK29 was replaced with the apramycin resistance cassette from pWH1266 (ATCC 77092), and the origin of replication was replaced with that from pFLP2^65^ to make it compatible with P-oriT-1. Primers for plasmid construction are listed in Table S9. Genetic complementation of mutant strains was then performed by cloning each gene of interest into the HindIII and NotI restriction sites of RFTool-1 (primers in Table S9). Site-directed mutagenesis was carried out by overlapping PCR using primers listed in Table S9.

## Supplementary information

Supplementary tables and figures

Table S2

Table S4

## Data Availability

Readers should address requests for materials, data and associated protocols to the corresponding author.

## References

[1] Liu P (2012). Complete genome sequence of *Klebsiella pneumoniae* subsp. *pneumoniae* HS11286, a multidrug-resistant strain isolated from human sputum. Journal of bacteriology.

[2] Bi D (2015). Mapping the resistance-associated mobilome of a carbapenem-resistant *Klebsiella pneumoniae* strain reveals insights into factors shaping these regions and facilitates generation of a ‘resistance-disarmed’model organism. Journal of Antimicrobial Chemotherapy.

[3] Qi Y (2010). ST11, the dominant clone of KPC-producing *Klebsiella pneumoniae* in China. Journal of Antimicrobial Chemotherapy.

[4] Munoz-Price LS (2013). Clinical epidemiology of the global expansion of *Klebsiella pneumoniae* carbapenemases. The Lancet infectious diseases.

[5] Chen L, Mathema B, Pitout JD, DeLeo FR, Kreiswirth BN (2014). Epidemic *Klebsiella pneumoniae* ST258 is a hybrid strain. mBio.

[6] Partridge SR (2011). Analysis of antibiotic resistance regions in Gram-negative bacteria. FEMS microbiology reviews.

[7] Frost LS, Leplae R, Summers AO, Toussaint A (2005). Mobile genetic elements: the agents of open source evolution. Nature reviews. Microbiology.

[8] Cheng H (2010). RmpA regulation of capsular polysaccharide biosynthesis in *Klebsiella pneumoniae* CG43. Journal of bacteriology.

[9] Bi D (2012). ICEberg: a web-based resource for integrative and conjugative elements found in Bacteria. Nucleic acids research.

[10] Liu M (2018). ICEberg 2.0: an updated database of bacterial integrative and conjugative elements. Nucleic acids research.

[11] Hochhut B (2006). Role of pathogenicity island‐associated integrases in the genome plasticity of uropathogenic *Escherichia coli* strain 536. Molecular microbiology.

[12] Carpenter MR, Rozovsky S, Boyd EF (2016). Pathogenicity island cross talk mediated by recombination directionality factors facilitates excision from the chromosome. Journal of bacteriology.

[13] Guédon G, Libante V, Coluzzi C, Payot S, Leblond-Bourget N (2017). The obscure world of integrative and mobilizable elements, highly widespread elements that pirate bacterial conjugative systems. Genes.

[14] Juhas M, Crook DW, Hood DW (2008). Type IV secretion systems: tools of bacterial horizontal gene transfer and virulence. Cellular microbiology.

[15] Juhas M (2009). Genomic islands: tools of bacterial horizontal gene transfer and evolution. Microbiol Rev.

[16] He J (2004). The broad host range pathogen *Pseudomonas aeruginosa* strain PA14 carries two pathogenicity islands harboring plant and animal virulence genes. Proceedings of the National Academy of Sciences.

[17] Larbig KD (2002). Gene Islands Integrated into tRNAGly Genes Confer Genome Diversity on a *Pseudomonas aeruginosa* Clone. Journal of bacteriology.

[18] Mavrodi DV, Loper JE, Paulsen IT, Thomashow LS (2009). Mobile genetic elements in the genome of the beneficial rhizobacterium *Pseudomonas fluorescens* Pf-5. BMC microbiology.

[19] Klockgether J, Reva O, Larbig K, Tummler B (2004). Sequence Analysis of the Mobile Genome Island pKLC102 of *Pseudomonas aeruginosa* C. Journal of Bacteriology.

[20] Roche D (2010). ICEEc2, a new integrative and conjugative element belonging to the pKLC102/PAGI-2 family, identified in *Escherichia coli* strain BEN374. Journal of bacteriology.

[21] Lin TL, Lee CZ, Hsieh PF, Tsai SF, Wang JT (2008). Characterization of integrative and conjugative element ICEKp1-associated genomic heterogeneity in a *Klebsiella pneumoniae* strain isolated from a primary liver abscess. Journal of bacteriology.

[22] Battle SE, Meyer F, Rello J, Kung VL, Hauser AR (2008). Hybrid pathogenicity island PAGI-5 contributes to the highly virulent phenotype of a *Pseudomonas aeruginosa* isolate in mammals. Journal of bacteriology.

[23] Klockgether J, Würdemann D, Reva O, Wiehlmann L, Tümmler B (2007). Diversity of the abundant pKLC102/PAGI-2 family of genomic islands in *Pseudomonas aeruginosa*. Journal of bacteriology.

[24] Wyres, K. L. *et al*. Identification of *Klebsiella* capsule synthesis loci from whole genome data. *Microbial genomics* **2** (2016). 10.1099/mgen.0.000102 PMC5359410 28348840

[25] Stoesser N (2013). Predicting antimicrobial susceptibilities for *Escherichia coli* and *Klebsiella pneumoniae* isolates using whole genomic sequence data. Journal of Antimicrobial Chemotherapy.

[26] Ko KS (2017). The contribution of capsule polysaccharide genes to virulence of *Klebsiella pneumoniae*. Virulence.

[27] Lam, M. M. *et al*. Genetic diversity, mobilisation and spread of the yersiniabactin-encoding mobile element ICEKp in *Klebsiella pneumoniae* populations. *Microbial genomics* **4** (2018). 10.1099/mgen.0.000196 PMC6202445 29985125

[28] Schmid B (2009). Role of cold shock proteins in growth of *Listeria monocytogenes* under cold and osmotic stress conditions. Appl Environ Microbiol.

[29] Bisht SC, Joshi GK, Mishra PK (2014). CspA encodes a major cold shock protein in Himalayan psychrotolerant *Pseudomonas* strains. Interdiscip Sci.

[30] Yamaguchi Y, Inouye M (2011). Regulation of growth and death in *Escherichia coli* by toxin-antitoxin systems. Nature reviews. Microbiology.

[31] Brantl S (2012). Bacterial type I toxin-antitoxin systems. RNA Biol.

[32] Mruk I, Kobayashi I (2014). To be or not to be: regulation of restriction-modification systems and other toxin-antitoxin systems. Nucleic acids research.

[33] McLaughlin HP, Caly DL, McCarthy Y, Ryan RP, Dow JM (2012). An orphan chemotaxis sensor regulates virulence and antibiotic tolerance in the human pathogen *Pseudomonas aeruginosa*. PloS one.

[34] de Paz HD (2005). Functional interactions between type IV secretion systems involved in DNA transfer and virulence. Microbiology.

[35] Xu J, Pei D, Nicholson A, Lan Y, Xia Q (2019). In Silico Identification of Three Types of Integrative and Conjugative Elements in *Elizabethkingia anophelis* Strains Isolated from around the World. mSphere.

[36] Zeng, L. *et al*. Genetic Characterization of a *blaVIM*–24-Carrying IncP-7β Plasmid p1160-VIM and a *blaVIM*–4-Harboring Integrative and Conjugative Element Tn6413 From Clinical *Pseudomonas aeruginosa*. *Frontiers in microbiology* **10** (2019). 10.3389/fmicb.2019.00213 PMC6399125 30863370

[37] Wozniak RA, Waldor MK (2010). Integrative and conjugative elements: mosaic mobile genetic elements enabling dynamic lateral gene flow. Nature Reviews Microbiology.

[38] Filloux A (2010). A variety of bacterial pili involved in horizontal gene transfer. Journal of bacteriology.

[39] Minoia M (2008). Stochasticity and bistability in horizontal transfer control of a genomic island in *Pseudomonas*. Proceedings of the National Academy of Sciences.

[40] Delavat F, Mitri S, Pelet S, van der Meer JR (2016). Highly variable individual donor cell fates characterize robust horizontal gene transfer of an integrative and conjugative element. Proceedings of the National Academy of Sciences.

[41] Rendueles O, de Sousa JAM, Bernheim A, Touchon M, Rocha EP (2018). Genetic exchanges are more frequent in bacteria encoding capsules. PLoS genetics.

[42] Gu D (2018). A fatal outbreak of ST11 carbapenem-resistant hypervirulent *Klebsiella pneumoniae* in a Chinese hospital: a molecular epidemiological study. The Lancet infectious diseases.

[43] Lam MM (2018). Population genomics of hypervirulent *Klebsiella pneumoniae* clonal-group 23 reveals early emergence and rapid global dissemination. Nature communications.

[44] Zechner EL, Lang S, Schildbach JF (2012). Assembly and mechanisms of bacterial type IV secretion machines. Philos Trans R Soc Lond B Biol Sci.

[45] Schroder G, Lanka E (2005). The mating pair formation system of conjugative plasmids-A versatile secretion machinery for transfer of proteins and DNA. Plasmid.

[46] Wallden K, Rivera-Calzada A, Waksman G (2010). Type IV secretion systems: versatility and diversity in function. Cellular microbiology.

[47] Cascales E, Christie PJ (2004). Definition of a Bacterial Type IV Secretion Pathway for a DNA Substrate. Science.

[48] Cascales E, Atmakuri K, Sarkar MK, Christie PJ (2013). DNA substrate-induced activation of the Agrobacterium VirB/VirD4 Type IV secretion system. Journal of bacteriology.

[49] Banta LM (2011). An Agrobacterium VirB10 mutation conferring a type IV secretion system gating defect. Journal of bacteriology.

[50] Atmakuri K, Cascales E, Christie PJ (2004). Energetic components VirD4, VirB11 and VirB4 mediate early DNA transfer reactions required for bacterial type IV secretion. Mol Microbiol.

[51] Lang S (2011). An activation domain of plasmid R1 TraI protein delineates stages of gene transfer initiation. Mol Microbiol.

[52] Gomis-Ruth FX (2001). The bacterial conjugation protein TrwB resembles ring helicases and F1-ATPase. Nature.

[53] Moncalian G (1999). Characterization of ATP and DNA binding activities of TrwB, the coupling protein essential in plasmid R388 conjugation. The Journal of Biological Chemistry.

[54] Hormaeche I (2006). The transmembrane domain provides nucleotide binding specificity to the bacterial conjugation protein TrwB. FEBS Lett.

[55] Aziz RK (2008). The RAST Server: rapid annotations using subsystems technology. BMC genomics.

[56] Kurtz S, Schleiermacher C (1999). REPuter: fast computation of maximal repeats in complete genomes. Bioinformatics (Oxford, England).

[57] Seemann T (2014). Prokka: rapid prokaryotic genome annotation. Bioinformatics.

[58] Page AJ (2015). Roary: rapid large-scale prokaryote pan genome analysis. Bioinformatics.

[59] Edgar RC (2004). MUSCLE: multiple sequence alignment with high accuracy and high throughput. Nucleic acids research.

[60] Stamatakis A (2014). RAxML version 8: a tool for phylogenetic analysis and post-analysis of large phylogenies. Bioinformatics.

[61] Kumar S, Stecher G, Li M, Knyaz C, Tamura K (2018). MEGA X: molecular evolutionary genetics analysis across computing platforms. Molecular biology and evolution.

[62] Schubert (2004). A novel integrative and conjugative element (ICE) of *Escherichia coli*: the putative progenitor of the *Yersinia* high-pathogenicity island. Molecular Microbiology.

[63] Lesic B, Rahme LG (2008). Use of the lambda Red recombinase system to rapidly generate mutants in *Pseudomonas aeruginosa*. BMC molecular biology.

[64] Wang RF, Kushner SR (1991). Construction of versatile low-copy-number vectors for cloning, sequencing and gene expression in *Escherichia coli*. Gene.

[65] Hoang TT, Karkhoff-Schweizer RR, Kutchma AJ, Schweizer HP (1998). A broad-host-range Flp-FRT recombination system for site-specific excision of chromosomally-located DNA sequences: application for isolation of unmarked *Pseudomonas aeruginosa* mutants. Gene.

[66] Carter MQ, Chen J, Lory S (2010). The *Pseudomonas aeruginosa* pathogenicity island PAPI-1 is transferred via a novel type IV pilus. Journal of bacteriology.

