# Supplementary material for: ICEKp2: description of an integrative and conjugative element in Klebsiella pneumoniae, co-occurring and interacting with ICEKp1
Source: Sci Rep. 2019 Sep 25;9:13892. doi: 10.1038/s41598-019-50456-x (PMC6761156; doi:10.1038/s41598-019-50456-x)
Supplement: Supplementary file 1 — Supplementary tables and figures [file 41598_2019_50456_MOESM1_ESM.pdf]

Title; ICEKp2: description of an integrative and conjugative element in *Klebsiella pneumoniae*, co-occurring and interacting with ICEKp1

Authors; Robeena Farzand, Kumar Rajakumar, Roxana Zamudio, Marco R Oggioni, Michael R Barer, Helen M O'Hare

Supplementary Tables and Figures

Table S1. In-silico characterisation and summary of ORFs found in ICEKp2.

Table S2. (Excel) Information on the genome sequences of *K. pneumoniae* isolates from the UK.

Table S3. Sequence types of *K. pneumoniae* isolates from US outbreak cross-referenced to table S4.

Table S4. (Excel) Information about the first 1000 whole genome sequences, retrieved from NCBI, used to investigate the occurrence and co-occurrence of ICEKp2 and ICEKp1.

Table S5 Information about representative strains of the 1000 genome (NCBI) analysis, used for phylogenetic analysis cross-referenced to Figure 3.

Table S6. Repeat sequences identified in ICEKp2 (candidates for oriT)

Table S7. Putative T4SS and relaxase identified in ICEKp2.

Table S8. Colony counts of donors and recipients before and after conjugation.

Table S9. Primers used in this study.

Figure S1. The occurrence of ICEKp1 and ICEKp2 in unsequenced of isolates of *K. pneumoniae* from Leicester, UK.

Figure S2. Gene disrupted ICEKp2 lacking *int2a* required a specific primer to amplify the attL site and this primer allowed efficient amplification of *attL* from chromosomal DNA of *K. pneumoniae* containing ICEKp2 ∆int2a.

Figure S3. Cloning whole ICEKp2 into the plasmid to test the presence of all possible origin of transfer in ICEKp2.

Figure S4. A variant of *mob2* with mutations in the ATP binding site (Walker A and B motifs) did not complement ICEKp1-driven plasmid transfer.

Figure S5. Validation of ICEKp1 driven conjugative mobilisation of P-oriT-1 to *E. coli*.

Table S1 *In silico* characterisation and summary of ORFs found in ICEKp2.

ORF	Strand	Amino acids	Homologue	Source	% identity	[b]Function of homologue
**ORF1**	-	315	AHM77645.1	*K. pneumoniae* 30684	100	DNA integration/recombination
**ORF2**	-	429	ESL95504.1	*K. pneumoniae* UCICRE8	99	Relaxase, PFGI-1 class
**ORF3**	-	327	EKF77242.1	*K. pneumoniae* Q3	99	ArdC
**ORF4**	-	202	WP_004151763.1	[a]*K. pneumoniae*	100	Hypothetical
**ORF5**	-	115	WP_004151762.1	[a]*K. pneumoniae*	100	Hypothetical
**ORF6**	-	132	SBL38293.1	*K. oxytoca*	98	ArdB
**ORF7**	-	92	WP_016809387.1	*Klebsiella* spp.	99	Hypothetical
**ORF8**	+	317	WP_004151759.1	[a]*K. pneumoniae*	100	Hypothetical
**ORF9**	-	297	EKF77241.1	*K. pneumoniae* Q3	99	Integrase
**ORF10**	-	92	GAS79470.1	*S. enterica*	86	Xre Family TF
**ORF11**	+	86	WP_016809394.1	*K. oxytoca*	93	Ner-like TF
**ORF12**	+	307	AAF30382.1	*Enterobacteria* phage HK022	99	IS903 Transposase
**ORF13**	+	154	AHM83223.1	*K. pneumoniae* 306601	100	Cold shock protein
**ORF14**	-	81	SBL38079.1	*K. oxytoca*	96	Xre Family TF
**ORF15**	+	83	EJJ32891.1	*K. pneumoniae* KPNIH4	83	Nlp TF
**ORF16**	+	99	EOR16801.1	*Klebsiella* spp.	100	Hypothetical
**ORF17**	-	509	WP_016809399.1	*K. oxytoca*	99	TraG
**ORF18**	-	113	WP_040236211.1	[a]*K. pneumoniae*	95	HP A9L51_14115
**ORF19**	-	473	EOR16806.1	*K. pneumoniae* UHKPC23	100	ICE protein, PFL_4711
**ORF20**	-	313	EOR16796.1	*K. pneumoniae* UHKPC23	100	ICE protein, PFL_4710
**ORF21**	-	131	EOR16813.1	*K. pneumoniae* UHKPC23	100	ICE protein, PFL_4709 family
**ORF22**	-	532	EOY67835.1	*K. pneumoniae* UHKPC40	100	HP H207_0366

**ORF23**	-	336	AHM77621.1	*K. pneumoniae* 30684	100	secA Preprotein Translocase
**ORF24**	-	143	EMI36389.1	*K. pneumoniae* VA360	100	Toxin SymE
**ORF25**	+	371	KLY12101.1	*K. oxytoca*	99	HP SK89_04460
**ORF26**	+	455	CDL12588.1	*K. pneumoniae* IS43	93	Glycosaminoglycan attachment protein
**ORF27**	-	44	WP_004217757.1	*Klebsiella* spp.	100	Hypothetical
**ORF28**	-	234	EJK38858.1	*K. pneumoniae* KPNIH23	100	Restriction endonuclease
**ORF29**	+	59	WP_004198954.1	*Klebsiella* spp.	100	Hypothetical
**ORF30**	-	924	EOZ52981.1	*K. pneumoniae* VAKPC309	98	CT ATPase, PFL_4706
**ORF31**	-	132	OBT34302.1	[a]*K. pneumoniae*	97	CT protein
**ORF32**	-	494	KTG59771.1	[a]*K. pneumoniae*	98	TrbI-like / PFL_4705
**ORF33**	-	281	EOR14930.1	*K. pneumoniae* UHKPC23	100	PFL_4704/TraK
**ORF34**	-	217	EOR14940.1	*K. pneumoniae* UHKPC23	100	ICE protein, PFL_4703
**ORF35**	-	123	EOR14950.1	*K. pneumoniae* UHKPC23	100	CT region protein, TIGR03750
**ORF36**	-	117	EOR14954.1	*K. pneumoniae* UHKPC23	100	ICE membrane protein, PFL_4702
**ORF37**	-	78	EOY67789.1	*K. pneumoniae* UHKPC40	100	ICE protein, PFL_4701
**ORF38**	-	105	SBL37582.1	*K. oxytoca*		ICE protein
**ORF39**	+	267	CFQ34406.1	*Y. enterocolitica*	67	Phosphoribosyl transferase
**ORF40**	+	158	WP_004150886.1	*Klebsiella* spp.	100	Hypothetical
**ORF41**	-	252	SBL37526.1	*K. oxytoca*	97	ICE membrane protein PFL_4697
**ORF42**	-	87	WP_004150888.1	*Klebsiella spp.*	93	Conserved Hypothetical protein
**ORF43**	-	699	EOY73211.1	*K. pneumoniae* UHKPC01	100	conjugative coupling factor TraD, PFGI-1 class
**ORF44**	-	170	EMI37003.1	*K. pneumoniae* VA360	100	ICE protein, PFL_4695
**ORF45**	-	189	SBL37436.1	*K. oxytoca*	99	Lytic murine transglycosylase
**ORF46**	-	205	SBL48986.1	*K. oxytoca*	98	ICE protein, PFL_4693 family
**ORF47**	-	246	CDK93076.1	*K. pneumoniae* IS33	100	Methyl-accepting chemotaxis protein
**ORF48**	-	189	SBL37378.1	*K. oxytoca*	99	Type IV B pilus protein

**ORF49**	-	150	EOZ31404.1	*K. pneumoniae* VAKPC269	100	PF12101 family protein
**ORF50**	-	240	EOR14922.1	*K. pneumoniae* UHKPC23	100	ICE protein, PFL_4669 family
**ORF51**	+	354	WP_004150897.1	*Klebsiella* spp.	100	TR, AbiEi antitoxin
**ORF52**	+	290	WP_004150898.1	*Klebsiella* spp.	100	Hypothetical
**ORF53**	-	251	ESL15922.1	*K. pneumoniae* BIDMC 41	99	Hypothetical L478_05391
**ORF54**	-	442	EPB38748.1	*K. pneumoniae* UHKPC32	100	WG repeat motif protein
**ORF55**	+	291	WP_004150901.1	*Klebsiella* spp.	100	Hypothetical
**ORF56**	-	405	EYB74595.1	*K. pneumoniae* Kb677	100	HTH / PFGI-1-like cluster
**ORF57**	-	194	CDL57295.1	*E. coli* ISC56	100	HP in PFGI-1-like cluster
**ORF58**	-	540	SBL37243.1	*K. oxytoca*	94	Transcriptional regulator/ParB family protein
**ORF59**	-	455	ESL95559.1	*K. pneumoniae* UCICRE8	99	Replicative DNA helicase
**ORF60**	-	128	WP_004150906.1	*Klebsiella* spp.	100	Hypothetical
**ORF61**	-	290	OBT34275.1	[a]*K. pneumoniae*	99	Chromosome-partitioning ATPase/ParA

[a] The identical sequence was present in more than one strain of K. pneumoniae.
[b] Abbreviations: TF = Transcriptional factor, CT = Conjugal Transfer, HTH = Helix turn helix domain (remove gene names, as it is confusing to explain some but not all of them)

Table S2. (Excel) Information about the genome sequences of *K. pneumoniae* isolates from the UK.

Table S3. Sequence types of *K. pneumoniae* isolates from US outbreak cross-referenced to table S4.

code	ST	gapA	infB	mdh	pgi	phoE	rpoB	tonB
CHS-02	1199	gapA(3)	infB(3)	mdh(1)	pgi(1)	phoE(1)	rpoB(67)	tonB(79)
CHS-03	258	gapA(3)	infB(3)	mdh(1)	pgi(1)	phoE(1)	rpoB(1)	tonB(79)
CHS-05	258	gapA(3)	infB(3)	mdh(1)	pgi(1)	phoE(1)	rpoB(1)	tonB(79)
CHS-06	258	gapA(3)	infB(3)	mdh(1)	pgi(1)	phoE(1)	rpoB(1)	tonB(79)
CHS-07	258	gapA(3)	infB(3)	mdh(1)	pgi(1)	phoE(1)	rpoB(1)	tonB(79)
CHS-08	258	gapA(3)	infB(3)	mdh(1)	pgi(1)	phoE(1)	rpoB(1)	tonB(79)
CHS-09	258	gapA(3)	infB(3)	mdh(1)	pgi(1)	phoE(1)	rpoB(1)	tonB(79)

CHS-11	258	gapA(3)	infB(3)	mdh(1)	pgi(1)	phoE(1)	rpoB(1)	tonB(79)
CHS-12	258	gapA(3)	infB(3)	mdh(1)	pgi(1)	phoE(1)	rpoB(1)	tonB(79)
CHS-13	258	gapA(3)	infB(3)	mdh(1)	pgi(1)	phoE(1)	rpoB(1)	tonB(79)
CHS-14	258	gapA(3)	infB(3)	mdh(1)	pgi(1)	phoE(1)	rpoB(1)	tonB(79)
CHS-16	258	gapA(3)	infB(3)	mdh(1)	pgi(1)	phoE(1)	rpoB(1)	tonB(79)
CHS-17	258	gapA(3)	infB(3)	mdh(1)	pgi(1)	phoE(1)	rpoB(1)	tonB(79)
CHS-18	258	gapA(3)	infB(3)	mdh(1)	pgi(1)	phoE(1)	rpoB(1)	tonB(79)
CHS-19	258	gapA(3)	infB(3)	mdh(1)	pgi(1)	phoE(1)	rpoB(1)	tonB(79)
CHS-20	258	gapA(3)	infB(3)	mdh(1)	pgi(1)	phoE(1)	rpoB(1)	tonB(79)
CHS-21	258	gapA(3)	infB(3)	mdh(1)	pgi(1)	phoE(1)	rpoB(1)	tonB(79)
CHS-22	258	gapA(3)	infB(3)	mdh(1)	pgi(1)	phoE(1)	rpoB(1)	tonB(79)
CHS-23	258	gapA(3)	infB(3)	mdh(1)	pgi(1)	phoE(1)	rpoB(1)	tonB(79)
CHS-25	258	gapA(3)	infB(3)	mdh(1)	pgi(1)	phoE(1)	rpoB(1)	tonB(79)
CHS-26	258	gapA(3)	infB(3)	mdh(1)	pgi(1)	phoE(1)	rpoB(1)	tonB(79)
CHS-27	258	gapA(3)	infB(3)	mdh(1)	pgi(1)	phoE(1)	rpoB(1)	tonB(79)
CHS-28	258	gapA(3)	infB(3)	mdh(1)	pgi(1)	phoE(1)	rpoB(1)	tonB(79)
CHS-29	258	gapA(3)	infB(3)	mdh(1)	pgi(1)	phoE(1)	rpoB(1)	tonB(79)
CHS-30	258	gapA(3)	infB(3)	mdh(1)	pgi(1)	phoE(1)	rpoB(1)	tonB(79)
CHS-31	258	gapA(3)	infB(3)	mdh(1)	pgi(1)	phoE(1)	rpoB(1)	tonB(79)
CHS-32	258	gapA(3)	infB(3)	mdh(1)	pgi(1)	phoE(1)	rpoB(1)	tonB(79)
CHS-33	258	gapA(3)	infB(3)	mdh(1)	pgi(1)	phoE(1)	rpoB(1)	tonB(79)
CHS-35	258	gapA(3)	infB(3)	mdh(1)	pgi(1)	phoE(1)	rpoB(1)	tonB(79)
CHS-36	258	gapA(3)	infB(3)	mdh(1)	pgi(1)	phoE(1)	rpoB(1)	tonB(79)
CHS-37	258	gapA(3)	infB(3)	mdh(1)	pgi(1)	phoE(1)	rpoB(1)	tonB(79)
CHS-38	258	gapA(3)	infB(3)	mdh(1)	pgi(1)	phoE(1)	rpoB(1)	tonB(79)
CHS-39	258	gapA(3)	infB(3)	mdh(1)	pgi(1)	phoE(1)	rpoB(1)	tonB(79)
CHS-40	258	gapA(3)	infB(3)	mdh(1)	pgi(1)	phoE(1)	rpoB(1)	tonB(79)

CHS-41	258	gapA(3)	infB(3)	mdh(1)	pgi(1)	phoE(1)	rpoB(1)	tonB(79)
CHS-42	258	gapA(3)	infB(3)	mdh(1)	pgi(1)	phoE(1)	rpoB(1)	tonB(79)
CHS-43	25	gapA(2)	infB(1)	mdh(1)	pgi(1)	phoE(10)	rpoB(4)	tonB(13)
CHS-44	258	gapA(3)	infB(3)	mdh(1)	pgi(1)	phoE(1)	rpoB(1)	tonB(79)
CHS-45	258	gapA(3)	infB(3)	mdh(1)	pgi(1)	phoE(1)	rpoB(1)	tonB(79)
CHS-46	258	gapA(3)	infB(3)	mdh(1)	pgi(1)	phoE(1)	rpoB(1)	tonB(79)
CHS-47	258	gapA(3)	infB(3)	mdh(1)	pgi(1)	phoE(1)	rpoB(1)	tonB(79)
CHS-48	25	gapA(2)	infB(1)	mdh(1)	pgi(1)	phoE(10)	rpoB(4)	tonB(13)
CHS-49	258	gapA(3)	infB(3)	mdh(1)	pgi(1)	phoE(1)	rpoB(1)	tonB(79)
CHS-50	258	gapA(3)	infB(3)	mdh(1)	pgi(1)	phoE(1)	rpoB(1)	tonB(79)
CHS-51	258	gapA(3)	infB(3)	mdh(1)	pgi(1)	phoE(1)	rpoB(1)	tonB(79)
CHS-52	258	gapA(3)	infB(3)	mdh(1)	pgi(1)	phoE(1)	rpoB(1)	tonB(79)
CHS-53	258	gapA(3)	infB(3)	mdh(1)	pgi(1)	phoE(1)	rpoB(1)	tonB(79)
CHS-54	258	gapA(3)	infB(3)	mdh(1)	pgi(1)	phoE(1)	rpoB(1)	tonB(79)
CHS-55	258	gapA(3)	infB(3)	mdh(1)	pgi(1)	phoE(1)	rpoB(1)	tonB(79)
CHS-56	258	gapA(3)	infB(3)	mdh(1)	pgi(1)	phoE(1)	rpoB(1)	tonB(79)
CHS-57	258	gapA(3)	infB(3)	mdh(1)	pgi(1)	phoE(1)	rpoB(1)	tonB(79)
CHS-58	258	gapA(3)	infB(3)	mdh(1)	pgi(1)	phoE(1)	rpoB(1)	tonB(79)
CHS-59	258	gapA(3)	infB(3)	mdh(1)	pgi(1)	phoE(1)	rpoB(1)	tonB(79)
CHS-60	258	gapA(3)	infB(3)	mdh(1)	pgi(1)	phoE(1)	rpoB(1)	tonB(79)
CHS-61	258	gapA(3)	infB(3)	mdh(1)	pgi(1)	phoE(1)	rpoB(1)	tonB(79)
CHS-62	258	gapA(3)	infB(3)	mdh(1)	pgi(1)	phoE(1)	rpoB(1)	tonB(79)
CHS-63	258	gapA(3)	infB(3)	mdh(1)	pgi(1)	phoE(1)	rpoB(1)	tonB(79)
CHS-64	258	gapA(3)	infB(3)	mdh(1)	pgi(1)	phoE(1)	rpoB(1)	tonB(79)
CHS-65	258	gapA(3)	infB(3)	mdh(1)	pgi(1)	phoE(1)	rpoB(1)	tonB(79)
CHS-66	14	gapA(1)	infB(6)	mdh(1)	pgi(1)	phoE(1)	rpoB(1)	tonB(1)
CHS-67	258	gapA(3)	infB(3)	mdh(1)	pgi(1)	phoE(1)	rpoB(1)	tonB(79)

CHS-70	258	gapA(3)	infB(3)	mdh(1)	pgi(1)	phoE(1)	rpoB(1)	tonB(79)
CHS-71	258	gapA(3)	infB(3)	mdh(1)	pgi(1)	phoE(1)	rpoB(1)	tonB(79)
CHS-72	258	gapA(3)	infB(3)	mdh(1)	pgi(1)	phoE(1)	rpoB(1)	tonB(79)
CHS-73	258	gapA(3)	infB(3)	mdh(1)	pgi(1)	phoE(1)	rpoB(1)	tonB(79)
CHS-74	258	gapA(3)	infB(3)	mdh(1)	pgi(1)	phoE(1)	rpoB(1)	tonB(79)
CHS-75	258	gapA(3)	infB(3)	mdh(1)	pgi(1)	phoE(1)	rpoB(1)	tonB(79)
CHS-76	258	gapA(3)	infB(3)	mdh(1)	pgi(1)	phoE(1)	rpoB(1)	tonB(79)
CHS-80	258	gapA(3)	infB(3)	mdh(1)	pgi(1)	phoE(1)	rpoB(1)	tonB(79)
CHS100	258	gapA(3)	infB(3)	mdh(1)	pgi(1)	phoE(1)	rpoB(1)	tonB(79)
CHS101	258	gapA(3)	infB(3)	mdh(1)	pgi(1)	phoE(1)	rpoB(1)	tonB(79)
CHS102	258	gapA(3)	infB(3)	mdh(1)	pgi(1)	phoE(1)	rpoB(1)	tonB(79)
CHS103	258	gapA(3)	infB(3)	mdh(1)	pgi(1)	phoE(1)	rpoB(1)	tonB(79)
CHS104	258	gapA(3)	infB(3)	mdh(1)	pgi(1)	phoE(1)	rpoB(1)	tonB(79)
CHS105	258	gapA(3)	infB(3)	mdh(1)	pgi(1)	phoE(1)	rpoB(1)	tonB(79)
CHS106	258	gapA(3)	infB(3)	mdh(1)	pgi(1)	phoE(1)	rpoB(1)	tonB(79)
CHS107	432	gapA(4)	infB(1)	mdh(1)	pgi(2)	phoE(7)	rpoB(4)	tonB(4)
CHS108	258	gapA(3)	infB(3)	mdh(1)	pgi(1)	phoE(1)	rpoB(1)	tonB(79)
CHS109	258	gapA(3)	infB(3)	mdh(1)	pgi(1)	phoE(1)	rpoB(1)	tonB(79)
CHS110	258	gapA(3)	infB(3)	mdh(1)	pgi(1)	phoE(1)	rpoB(1)	tonB(79)
CHS111	258	gapA(3)	infB(3)	mdh(1)	pgi(1)	phoE(1)	rpoB(1)	tonB(79)
CHS112	15	gapA(1)	infB(1)	mdh(1)	pgi(1)	phoE(1)	rpoB(1)	tonB(1)
CHS114	258	gapA(3)	infB(3)	mdh(1)	pgi(1)	phoE(1)	rpoB(1)	tonB(79)
CHS115	258	gapA(3)	infB(3)	mdh(1)	pgi(1)	phoE(1)	rpoB(1)	tonB(79)
CHS116	258	gapA(3)	infB(3)	mdh(1)	pgi(1)	phoE(1)	rpoB(1)	tonB(79)
CHS117	258	gapA(3)	infB(3)	mdh(1)	pgi(1)	phoE(1)	rpoB(1)	tonB(79)
CHS118	258	gapA(3)	infB(3)	mdh(1)	pgi(1)	phoE(1)	rpoB(1)	tonB(79)
CHS119	258	gapA(3)	infB(3)	mdh(1)	pgi(1)	phoE(1)	rpoB(1)	tonB(79)

CHS120	258	gapA(3)	infB(3)	mdh(1)	pgi(1)	phoE(1)	rpoB(1)	tonB(79)
CHS121	405	gapA(2)	infB(1)	mdh(62)	pgi(3)	phoE(10)	rpoB(4)	tonB(110)
CHS122	258	gapA(3)	infB(3)	mdh(1)	pgi(1)	phoE(1)	rpoB(1)	tonB(79)
CHS123	258	gapA(3)	infB(3)	mdh(1)	pgi(1)	phoE(1)	rpoB(1)	tonB(79)
CHS124	258	gapA(3)	infB(3)	mdh(1)	pgi(1)	phoE(1)	rpoB(1)	tonB(79)
CHS125	258	gapA(3)	infB(3)	mdh(1)	pgi(1)	phoE(1)	rpoB(1)	tonB(79)
CHS126	258	gapA(3)	infB(3)	mdh(1)	pgi(1)	phoE(1)	rpoB(1)	tonB(79)
CHS127	258	gapA(3)	infB(3)	mdh(1)	pgi(1)	phoE(1)	rpoB(1)	tonB(79)
CHS128	258	gapA(3)	infB(3)	mdh(1)	pgi(1)	phoE(1)	rpoB(1)	tonB(79)
CHS129	258	gapA(3)	infB(3)	mdh(1)	pgi(1)	phoE(1)	rpoB(1)	tonB(79)
CHS130	258	gapA(3)	infB(3)	mdh(1)	pgi(1)	phoE(1)	rpoB(1)	tonB(79)
CHS131	258	gapA(3)	infB(3)	mdh(1)	pgi(1)	phoE(1)	rpoB(1)	tonB(79)
CHS132	258	gapA(3)	infB(3)	mdh(1)	pgi(1)	phoE(1)	rpoB(1)	tonB(79)
CHS133	258	gapA(3)	infB(3)	mdh(1)	pgi(1)	phoE(1)	rpoB(1)	tonB(79)
CHS134	258	gapA(3)	infB(3)	mdh(1)	pgi(1)	phoE(1)	rpoB(1)	tonB(79)
CHS135	258	gapA(3)	infB(3)	mdh(1)	pgi(1)	phoE(1)	rpoB(1)	tonB(79)
CHS136	new	gapA(2)	infB(6)	mdh(1)	pgi(~42)	phoE(10)	rpoB(4)	tonB(5)
CHS138	258	gapA(3)	infB(3)	mdh(1)	pgi(1)	phoE(1)	rpoB(1)	tonB(79)
CHS139	258	gapA(3)	infB(3)	mdh(1)	pgi(1)	phoE(1)	rpoB(1)	tonB(79)
CHS140	14	gapA(1)	infB(6)	mdh(1)	pgi(1)	phoE(1)	rpoB(1)	tonB(1)
CHS141	258	gapA(3)	infB(3)	mdh(1)	pgi(1)	phoE(1)	rpoB(1)	tonB(79)
CHS142	258	gapA(3)	infB(3)	mdh(1)	pgi(1)	phoE(1)	rpoB(1)	tonB(79)
CHS144	258	gapA(3)	infB(3)	mdh(1)	pgi(1)	phoE(1)	rpoB(1)	tonB(79)
CHS145	1411	gapA(2)	infB(1)	mdh(20)	pgi(2)	phoE(1)	rpoB(1)	tonB(10)
CHS146	29	gapA(2)	infB(3)	mdh(2)	pgi(2)	phoE(6)	rpoB(4)	tonB(4)
CHS147	258	gapA(3)	infB(3)	mdh(1)	pgi(1)	phoE(1)	rpoB(1)	tonB(79)
CHS148	258	gapA(3)	infB(3)	mdh(1)	pgi(1)	phoE(1)	rpoB(1)	tonB(79)

CHS149	258	gapA(3)	infB(3)	mdh(1)	pgi(1)	phoE(1)	rpoB(1)	tonB(79)
CHS150	258	gapA(3)	infB(3)	mdh(1)	pgi(1)	phoE(1)	rpoB(1)	tonB(79)
CHS151	258	gapA(3)	infB(3)	mdh(1)	pgi(1)	phoE(1)	rpoB(1)	tonB(79)
CHS152	258	gapA(3)	infB(3)	mdh(1)	pgi(1)	phoE(1)	rpoB(1)	tonB(79)
CHS154	258	gapA(3)	infB(3)	mdh(1)	pgi(1)	phoE(1)	rpoB(1)	tonB(79)
CHS155	258	gapA(3)	infB(3)	mdh(1)	pgi(1)	phoE(1)	rpoB(1)	tonB(79)
CHS156	258	gapA(3)	infB(3)	mdh(1)	pgi(1)	phoE(1)	rpoB(1)	tonB(79)
CHS157	258	gapA(3)	infB(3)	mdh(1)	pgi(1)	phoE(1)	rpoB(1)	tonB(79)
CHS158	258	gapA(3)	infB(3)	mdh(1)	pgi(1)	phoE(1)	rpoB(1)	tonB(79)
CHS159	158	gapA(1)	infB(4)	mdh(35)	pgi(1)	phoE(10)	rpoB(25)	tonB(14)
CHS160	258	gapA(3)	infB(3)	mdh(1)	pgi(1)	phoE(1)	rpoB(1)	tonB(79)
CHS161	29	gapA(2)	infB(3)	mdh(2)	pgi(2)	phoE(6)	rpoB(4)	tonB(4)
CHS162	new	gapA(3)	infB(1)	mdh(2)	pgi(1)	phoE(9)	rpoB(4)	tonB(8)
CHS163	258	gapA(3)	infB(3)	mdh(1)	pgi(1)	phoE(1)	rpoB(1)	tonB(79)
CHS164	258	gapA(3)	infB(3)	mdh(1)	pgi(1)	phoE(1)	rpoB(1)	tonB(79)
CHS165	258	gapA(3)	infB(3)	mdh(1)	pgi(1)	phoE(1)	rpoB(1)	tonB(79)
CHS166	258	gapA(3)	infB(3)	mdh(1)	pgi(1)	phoE(1)	rpoB(1)	tonB(79)
CHS167	258	gapA(3)	infB(3)	mdh(1)	pgi(1)	phoE(1)	rpoB(1)	tonB(79)
CHS168	258	gapA(3)	infB(3)	mdh(1)	pgi(1)	phoE(1)	rpoB(1)	tonB(79)
CHS170	258	gapA(3)	infB(3)	mdh(1)	pgi(1)	phoE(1)	rpoB(1)	tonB(79)
CHS171	258	gapA(3)	infB(3)	mdh(1)	pgi(1)	phoE(1)	rpoB(1)	tonB(79)
CHS172	258	gapA(3)	infB(3)	mdh(1)	pgi(1)	phoE(1)	rpoB(1)	tonB(79)
CHS173	258	gapA(3)	infB(3)	mdh(1)	pgi(1)	phoE(1)	rpoB(1)	tonB(79)
CHS174	new	gapA(3)	infB(1)	mdh(2)	pgi(~3)	phoE(10)	rpoB(5)	tonB(19)
CHS175	14	gapA(1)	infB(6)	mdh(1)	pgi(1)	phoE(1)	rpoB(1)	tonB(1)
CHS176	258	gapA(3)	infB(3)	mdh(1)	pgi(1)	phoE(1)	rpoB(1)	tonB(79)
CHS177	258	gapA(3)	infB(3)	mdh(1)	pgi(1)	phoE(1)	rpoB(1)	tonB(79)

CHS178	258	gapA(3)	infB(3)	mdh(1)	pgi(1)	phoE(1)	rpoB(1)	tonB(79)
CHS181	258	gapA(3)	infB(3)	mdh(1)	pgi(1)	phoE(1)	rpoB(1)	tonB(79)
CHS182	258	gapA(3)	infB(3)	mdh(1)	pgi(1)	phoE(1)	rpoB(1)	tonB(79)
CHS183	258	gapA(3)	infB(3)	mdh(1)	pgi(1)	phoE(1)	rpoB(1)	tonB(79)
CHS184	258	gapA(3)	infB(3)	mdh(1)	pgi(1)	phoE(1)	rpoB(1)	tonB(79)
CHS185	258	gapA(3)	infB(3)	mdh(1)	pgi(1)	phoE(1)	rpoB(1)	tonB(79)
CHS186	258	gapA(3)	infB(3)	mdh(1)	pgi(1)	phoE(1)	rpoB(1)	tonB(79)
CHS187	258	gapA(3)	infB(3)	mdh(1)	pgi(1)	phoE(1)	rpoB(1)	tonB(79)
CHS189	258	gapA(3)	infB(3)	mdh(1)	pgi(1)	phoE(1)	rpoB(1)	tonB(79)
CHS191	258	gapA(3)	infB(3)	mdh(1)	pgi(1)	phoE(1)	rpoB(1)	tonB(79)
CHS192	258	gapA(3)	infB(3)	mdh(1)	pgi(1)	phoE(1)	rpoB(1)	tonB(79)
CHS194	258	gapA(3)	infB(3)	mdh(1)	pgi(1)	phoE(1)	rpoB(1)	tonB(79)
CHS195	258	gapA(3)	infB(3)	mdh(1)	pgi(1)	phoE(1)	rpoB(1)	tonB(79)
CHS196	258	gapA(3)	infB(3)	mdh(1)	pgi(1)	phoE(1)	rpoB(1)	tonB(79)
CHS197	258	gapA(3)	infB(3)	mdh(1)	pgi(1)	phoE(1)	rpoB(1)	tonB(79)
CHS198	258	gapA(3)	infB(3)	mdh(1)	pgi(1)	phoE(1)	rpoB(1)	tonB(79)
CHS200	258	gapA(3)	infB(3)	mdh(1)	pgi(1)	phoE(1)	rpoB(1)	tonB(79)
CHS201	new	gapA(~3)	infB(1)	mdh(1)	pgi(3)	phoE(4)	rpoB(28)	tonB(39)
CHS202	258	gapA(3)	infB(3)	mdh(1)	pgi(1)	phoE(1)	rpoB(1)	tonB(79)
CHS205	258	gapA(3)	infB(3)	mdh(1)	pgi(1)	phoE(1)	rpoB(1)	tonB(79)
CHS206	2121	gapA(3)	infB(1)	mdh(1)	pgi(4)	phoE(7)	rpoB(4)	tonB(13)
CHS207	258	gapA(3)	infB(3)	mdh(1)	pgi(1)	phoE(1)	rpoB(1)	tonB(79)
CHS208	11	gapA(3)	infB(3)	mdh(1)	pgi(1)	phoE(1)	rpoB(1)	tonB(4)
CHS209	258	gapA(3)	infB(3)	mdh(1)	pgi(1)	phoE(1)	rpoB(1)	tonB(79)
CHS210	258	gapA(3)	infB(3)	mdh(1)	pgi(1)	phoE(1)	rpoB(1)	tonB(79)
CHS211	new	gapA(3)	infB(3)	mdh(1)	pgi(1)	phoE(1)	rpoB(67)	tonB(4)
CHS212	258	gapA(3)	infB(3)	mdh(1)	pgi(1)	phoE(1)	rpoB(1)	tonB(79)

CHS213	258	gapA(3)	infB(3)	mdh(1)	pgi(1)	phoE(1)	rpoB(1)	tonB(79)
CHS214	258	gapA(3)	infB(3)	mdh(1)	pgi(1)	phoE(1)	rpoB(1)	tonB(79)
CHS215	258	gapA(3)	infB(3)	mdh(1)	pgi(1)	phoE(1)	rpoB(1)	tonB(79)
CHS216	258	gapA(3)	infB(3)	mdh(1)	pgi(1)	phoE(1)	rpoB(1)	tonB(79)
CHS217	258	gapA(3)	infB(3)	mdh(1)	pgi(1)	phoE(1)	rpoB(1)	tonB(79)
CHS218	258	gapA(3)	infB(3)	mdh(1)	pgi(1)	phoE(1)	rpoB(1)	tonB(79)
CHS219	258	gapA(3)	infB(3)	mdh(1)	pgi(1)	phoE(1)	rpoB(1)	tonB(79)
CHS220	258	gapA(3)	infB(3)	mdh(1)	pgi(1)	phoE(1)	rpoB(1)	tonB(79)
CHS221	258	gapA(3)	infB(3)	mdh(1)	pgi(1)	phoE(1)	rpoB(1)	tonB(79)
CHS223	258	gapA(3)	infB(3)	mdh(1)	pgi(1)	phoE(1)	rpoB(1)	tonB(79)
CHS224	258	gapA(3)	infB(3)	mdh(1)	pgi(1)	phoE(1)	rpoB(1)	tonB(79)
CHS225	258	gapA(3)	infB(3)	mdh(1)	pgi(1)	phoE(1)	rpoB(1)	tonB(79)
CHS226	258	gapA(3)	infB(3)	mdh(1)	pgi(1)	phoE(1)	rpoB(1)	tonB(79)
CHS228	258	gapA(3)	infB(3)	mdh(1)	pgi(1)	phoE(1)	rpoB(1)	tonB(79)
CHS229	11	gapA(3)	infB(3)	mdh(1)	pgi(1)	phoE(1)	rpoB(1)	tonB(4)
CHS230	258	gapA(3)	infB(3)	mdh(1)	pgi(1)	phoE(1)	rpoB(1)	tonB(79)
CHS231	258	gapA(3)	infB(3)	mdh(1)	pgi(1)	phoE(1)	rpoB(1)	tonB(79)
CHS232	258	gapA(3)	infB(3)	mdh(1)	pgi(1)	phoE(1)	rpoB(1)	tonB(79)
CHS234	258	gapA(3)	infB(3)	mdh(1)	pgi(1)	phoE(1)	rpoB(1)	tonB(79)
CHS235	258	gapA(3)	infB(3)	mdh(1)	pgi(1)	phoE(1)	rpoB(1)	tonB(79)
CHS236	258	gapA(3)	infB(3)	mdh(1)	pgi(1)	phoE(1)	rpoB(1)	tonB(79)
CHS237	258	gapA(3)	infB(3)	mdh(1)	pgi(1)	phoE(1)	rpoB(1)	tonB(79)
CHS238	258	gapA(3)	infB(3)	mdh(1)	pgi(1)	phoE(1)	rpoB(1)	tonB(79)
CHS239	258	gapA(3)	infB(3)	mdh(1)	pgi(1)	phoE(1)	rpoB(1)	tonB(79)
CHS240	258	gapA(3)	infB(3)	mdh(1)	pgi(1)	phoE(1)	rpoB(1)	tonB(79)
CHS81	258	gapA(3)	infB(3)	mdh(1)	pgi(1)	phoE(1)	rpoB(1)	tonB(79)
CHS82	258	gapA(3)	infB(3)	mdh(1)	pgi(1)	phoE(1)	rpoB(1)	tonB(79)

CHS83	258	gapA(3)	infB(3)	mdh(1)	pgi(1)	phoE(1)	rpoB(1)	tonB(79)
CHS84	258	gapA(3)	infB(3)	mdh(1)	pgi(1)	phoE(1)	rpoB(1)	tonB(79)
CHS85	258	gapA(3)	infB(3)	mdh(1)	pgi(1)	phoE(1)	rpoB(1)	tonB(79)
CHS86	11	gapA(3)	infB(3)	mdh(1)	pgi(1)	phoE(1)	rpoB(1)	tonB(4)
CHS87	258	gapA(3)	infB(3)	mdh(1)	pgi(1)	phoE(1)	rpoB(1)	tonB(79)
CHS88	258	gapA(3)	infB(3)	mdh(1)	pgi(1)	phoE(1)	rpoB(1)	tonB(79)
CHS89	258	gapA(3)	infB(3)	mdh(1)	pgi(1)	phoE(1)	rpoB(1)	tonB(79)
CHS90	258	gapA(3)	infB(3)	mdh(1)	pgi(1)	phoE(1)	rpoB(1)	tonB(79)
CHS91	258	gapA(3)	infB(3)	mdh(1)	pgi(1)	phoE(1)	rpoB(1)	tonB(79)
CHS92	258	gapA(3)	infB(3)	mdh(1)	pgi(1)	phoE(1)	rpoB(1)	tonB(79)
CHS93	11	gapA(3)	infB(3)	mdh(1)	pgi(1)	phoE(1)	rpoB(1)	tonB(4)
CHS94	258	gapA(3)	infB(3)	mdh(1)	pgi(1)	phoE(1)	rpoB(1)	tonB(79)
CHS95	258	gapA(3)	infB(3)	mdh(1)	pgi(1)	phoE(1)	rpoB(1)	tonB(79)
CHS96	258	gapA(3)	infB(3)	mdh(1)	pgi(1)	phoE(1)	rpoB(1)	tonB(79)
CHS97	258	gapA(3)	infB(3)	mdh(1)	pgi(1)	phoE(1)	rpoB(1)	tonB(79)
CHS98	258	gapA(3)	infB(3)	mdh(1)	pgi(1)	phoE(1)	rpoB(1)	tonB(79)
CHS99	258	gapA(3)	infB(3)	mdh(1)	pgi(1)	phoE(1)	rpoB(1)	tonB(79)
UCI-17	437	gapA(3)	infB(3)	mdh(1)	pgi(1)	phoE(1)	rpoB(1)	tonB(31)
UCI-19	258	gapA(3)	infB(3)	mdh(1)	pgi(1)	phoE(1)	rpoB(1)	tonB(79)
UCI-20	new	gapA(2)	infB(~1)	mdh(2)	pgi(1)	phoE(7)	rpoB(1)	tonB(81)
UCI-21	258	gapA(3)	infB(3)	mdh(1)	pgi(1)	phoE(1)	rpoB(1)	tonB(79)
UCI-22	258	gapA(3)	infB(3)	mdh(1)	pgi(1)	phoE(1)	rpoB(1)	tonB(79)
UCI-25	873	gapA(14)	infB(1)	mdh(2)	pgi(1)	phoE(7)	rpoB(4)	tonB(182)
UCI-26	new	gapA(4)	infB(18)	mdh(5)	pgi(1)	phoE(26)	rpoB(8)	tonB(13)
UCI-33	258	gapA(3)	infB(3)	mdh(1)	pgi(1)	phoE(1)	rpoB(1)	tonB(79)
UCI-34	1444	gapA(2)	infB(3)	mdh(6)	pgi(1)	phoE(17)	rpoB(7)	tonB(4)
UCI-37	258	gapA(3)	infB(3)	mdh(1)	pgi(1)	phoE(1)	rpoB(1)	tonB(79)

UCI-38	258	gapA(3)	infB(3)	mdh(1)	pgi(1)	phoE(1)	rpoB(1)	tonB(79)
UCI-41	258	gapA(3)	infB(3)	mdh(1)	pgi(1)	phoE(1)	rpoB(1)	tonB(79)
UCI-42	86	gapA(9)	infB(4)	mdh(2)	pgi(1)	phoE(1)	rpoB(1)	tonB(27)
UCI-43	258	gapA(3)	infB(3)	mdh(1)	pgi(1)	phoE(1)	rpoB(1)	tonB(79)
UCI-55	258	gapA(3)	infB(3)	mdh(1)	pgi(1)	phoE(1)	rpoB(1)	tonB(79)
UCI-56	678	gapA(2)	infB(3)	mdh(1)	pgi(1)	phoE(109)	rpoB(56)	tonB(18)
UCI-59	258	gapA(3)	infB(3)	mdh(1)	pgi(1)	phoE(1)	rpoB(1)	tonB(79)
UCI-60	15	gapA(1)	infB(1)	mdh(1)	pgi(1)	phoE(1)	rpoB(1)	tonB(1)
UCI-61	258	gapA(3)	infB(3)	mdh(1)	pgi(1)	phoE(1)	rpoB(1)	tonB(79)
UCI-62	34	gapA(2)	infB(3)	mdh(6)	pgi(1)	phoE(9)	rpoB(7)	tonB(4)
UCI-63	258	gapA(3)	infB(3)	mdh(1)	pgi(1)	phoE(1)	rpoB(1)	tonB(79)
UCI-64	new	gapA(4)	infB(1)	mdh(2)	pgi(80)	phoE(4)	rpoB(4)	tonB(~2)
UCI-67	258	gapA(3)	infB(3)	mdh(1)	pgi(1)	phoE(1)	rpoB(1)	tonB(79)
UCI-68	37	gapA(2)	infB(9)	mdh(2)	pgi(1)	phoE(13)	rpoB(1)	tonB(16)
UCI69	258	gapA(3)	infB(3)	mdh(1)	pgi(1)	phoE(1)	rpoB(1)	tonB(79)
UCI70	997	gapA(4)	infB(6)	mdh(1)	pgi(1)	phoE(1)	rpoB(28)	tonB(34)
UCI75	258	gapA(3)	infB(3)	mdh(1)	pgi(1)	phoE(1)	rpoB(1)	tonB(79)
UCI76	322	gapA(2)	infB(1)	mdh(5)	pgi(1)	phoE(26)	rpoB(4)	tonB(12)
UCI81	258	gapA(3)	infB(3)	mdh(1)	pgi(1)	phoE(1)	rpoB(1)	tonB(79)
UCI82	45	gapA(2)	infB(1)	mdh(1)	pgi(6)	phoE(7)	rpoB(1)	tonB(12)
UCI91	258	gapA(3)	infB(3)	mdh(1)	pgi(1)	phoE(1)	rpoB(1)	tonB(79)
UCI92	107	gapA(2)	infB(1)	mdh(2)	pgi(17)	phoE(27)	rpoB(1)	tonB(39)
UCI93	258	gapA(3)	infB(3)	mdh(1)	pgi(1)	phoE(1)	rpoB(1)	tonB(79)
UCI94	277	gapA(3)	infB(1)	mdh(1)	pgi(1)	phoE(1)	rpoB(1)	tonB(43)
UCI95	258	gapA(3)	infB(3)	mdh(1)	pgi(1)	phoE(1)	rpoB(1)	tonB(79)
UCI96	15	gapA(1)	infB(1)	mdh(1)	pgi(1)	phoE(1)	rpoB(1)	tonB(1)
UCICRE-1	258	gapA(3)	infB(3)	mdh(1)	pgi(1)	phoE(1)	rpoB(1)	tonB(79)

UCICRE-13	258	gapA(3)	infB(3)	mdh(1)	pgi(1)	phoE(1)	rpoB(1)	tonB(79)
UCICRE-2	37	gapA(2)	infB(9)	mdh(2)	pgi(1)	phoE(13)	rpoB(1)	tonB(16)
UCICRE-4	14	gapA(1)	infB(6)	mdh(1)	pgi(1)	phoE(1)	rpoB(1)	tonB(1)
UCICRE-6	86	gapA(9)	infB(4)	mdh(2)	pgi(1)	phoE(1)	rpoB(1)	tonB(27)
UCICRE-7	17	gapA(2)	infB(1)	mdh(1)	pgi(1)	phoE(4)	rpoB(4)	tonB(4)
UCICRE-8	1198	gapA(2)	infB(9)	mdh(2)	pgi(1)	phoE(9)	rpoB(1)	tonB(4)
UCLAOXA232KP	16	gapA(2)	infB(1)	mdh(2)	pgi(1)	phoE(4)	rpoB(4)	tonB(4)
UHKPC-52	258	gapA(3)	infB(3)	mdh(1)	pgi(1)	phoE(1)	rpoB(1)	tonB(79)
UHKPC01	258	gapA(3)	infB(3)	mdh(1)	pgi(1)	phoE(1)	rpoB(1)	tonB(79)
UHKPC02	258	gapA(3)	infB(3)	mdh(1)	pgi(1)	phoE(1)	rpoB(1)	tonB(79)
UHKPC05	37	gapA(2)	infB(9)	mdh(2)	pgi(1)	phoE(13)	rpoB(1)	tonB(16)
UHKPC06	258	gapA(3)	infB(3)	mdh(1)	pgi(1)	phoE(1)	rpoB(1)	tonB(79)
UHKPC07	258	gapA(3)	infB(3)	mdh(1)	pgi(1)	phoE(1)	rpoB(1)	tonB(79)
UHKPC09	258	gapA(3)	infB(3)	mdh(1)	pgi(1)	phoE(1)	rpoB(1)	tonB(79)
UHKPC17	258	gapA(3)	infB(3)	mdh(1)	pgi(1)	phoE(1)	rpoB(1)	tonB(79)
UHKPC179	45	gapA(2)	infB(1)	mdh(1)	pgi(6)	phoE(7)	rpoB(1)	tonB(12)
UHKPC18	258	gapA(3)	infB(3)	mdh(1)	pgi(1)	phoE(1)	rpoB(1)	tonB(79)
UHKPC22	258	gapA(3)	infB(3)	mdh(1)	pgi(1)	phoE(1)	rpoB(1)	tonB(79)
UHKPC23	1272	gapA(2)	infB(1)	mdh(2)	pgi(1)	phoE(7)	rpoB(86)	tonB(7)
UHKPC24	258	gapA(3)	infB(3)	mdh(1)	pgi(1)	phoE(1)	rpoB(1)	tonB(79)
UHKPC26	258	gapA(3)	infB(3)	mdh(1)	pgi(1)	phoE(1)	rpoB(1)	tonB(79)
UHKPC27	258	gapA(3)	infB(3)	mdh(1)	pgi(1)	phoE(1)	rpoB(1)	tonB(79)
UHKPC28	258	gapA(3)	infB(3)	mdh(1)	pgi(1)	phoE(1)	rpoB(1)	tonB(79)
UHKPC29	258	gapA(3)	infB(3)	mdh(1)	pgi(1)	phoE(1)	rpoB(1)	tonB(79)
UHKPC31	258	gapA(3)	infB(3)	mdh(1)	pgi(1)	phoE(1)	rpoB(1)	tonB(79)
UHKPC33	258	gapA(3)	infB(3)	mdh(1)	pgi(1)	phoE(1)	rpoB(1)	tonB(79)
UHKPC40	258	gapA(3)	infB(3)	mdh(1)	pgi(1)	phoE(1)	rpoB(1)	tonB(79)

UHKPC45	258	gapA(3)	infB(3)	mdh(1)	pgi(1)	phoE(1)	rpoB(1)	tonB(79)
UHKPC47	258	gapA(3)	infB(3)	mdh(1)	pgi(1)	phoE(1)	rpoB(1)	tonB(79)
UHKPC48	258	gapA(3)	infB(3)	mdh(1)	pgi(1)	phoE(1)	rpoB(1)	tonB(79)
UHKPC57	1271	gapA(2)	infB(3)	mdh(2)	pgi(2)	phoE(4)	rpoB(4)	tonB(4)
UHKPC59	258	gapA(3)	infB(3)	mdh(1)	pgi(1)	phoE(1)	rpoB(1)	tonB(79)
UHKPC61	258	gapA(3)	infB(3)	mdh(1)	pgi(1)	phoE(1)	rpoB(1)	tonB(79)
UHKPC67	15	gapA(1)	infB(1)	mdh(1)	pgi(1)	phoE(1)	rpoB(1)	tonB(1)
UHKPC69	258	gapA(3)	infB(3)	mdh(1)	pgi(1)	phoE(1)	rpoB(1)	tonB(79)
UHKPC81	258	gapA(3)	infB(3)	mdh(1)	pgi(1)	phoE(1)	rpoB(1)	tonB(79)
UHKPC96	258	gapA(3)	infB(3)	mdh(1)	pgi(1)	phoE(1)	rpoB(1)	tonB(79)
UMNturkey9	45	gapA(2)	infB(1)	mdh(1)	pgi(6)	phoE(7)	rpoB(1)	tonB(12)
Urmite	67	gapA(2)	infB(1)	mdh(9)	pgi(1)	phoE(15)	rpoB(5)	tonB(28)
VA360	14	gapA(1)	infB(6)	mdh(1)	pgi(1)	phoE(1)	rpoB(1)	tonB(1)
VAKPC252	258	gapA(3)	infB(3)	mdh(1)	pgi(1)	phoE(1)	rpoB(1)	tonB(79)
VAKPC254	258	gapA(3)	infB(3)	mdh(1)	pgi(1)	phoE(1)	rpoB(1)	tonB(79)
VAKPC269	258	gapA(3)	infB(3)	mdh(1)	pgi(1)	phoE(1)	rpoB(1)	tonB(79)
VAKPC270	258	gapA(3)	infB(3)	mdh(1)	pgi(1)	phoE(1)	rpoB(1)	tonB(79)
VAKPC276	258	gapA(3)	infB(3)	mdh(1)	pgi(1)	phoE(1)	rpoB(1)	tonB(79)
VAKPC278	258	gapA(3)	infB(3)	mdh(1)	pgi(1)	phoE(1)	rpoB(1)	tonB(79)
VAKPC280	258	gapA(3)	infB(3)	mdh(1)	pgi(1)	phoE(1)	rpoB(1)	tonB(79)
VAKPC297	258	gapA(3)	infB(3)	mdh(1)	pgi(1)	phoE(1)	rpoB(1)	tonB(79)
VAKPC309	258	gapA(3)	infB(3)	mdh(1)	pgi(1)	phoE(1)	rpoB(1)	tonB(79)
W14	1536	gapA(2)	infB(1)	mdh(2)	pgi(37)	phoE(45)	rpoB(4)	tonB(9)
WCHKP020030	11	gapA(3)	infB(3)	mdh(1)	pgi(1)	phoE(1)	rpoB(1)	tonB(4)
WCHKP040035	11	gapA(3)	infB(3)	mdh(1)	pgi(1)	phoE(1)	rpoB(1)	tonB(4)
WCHKP095845	1	gapA(4)	infB(4)	mdh(1)	pgi(1)	phoE(7)	rpoB(4)	tonB(10)
WCHKP13F2	36	gapA(2)	infB(1)	mdh(2)	pgi(1)	phoE(7)	rpoB(1)	tonB(7)

WCHKP2	11	gapA(3)	infB(3)	mdh(1)	pgi(1)	phoE(1)	rpoB(1)	tonB(4)
WCHKP3	11	gapA(3)	infB(3)	mdh(1)	pgi(1)	phoE(1)	rpoB(1)	tonB(4)
WCHKP34	273	gapA(3)	infB(4)	mdh(6)	pgi(1)	phoE(7)	rpoB(4)	tonB(4)
WCHKP36	11	gapA(3)	infB(3)	mdh(1)	pgi(1)	phoE(1)	rpoB(1)	tonB(4)
WCHKP649	11	gapA(3)	infB(3)	mdh(1)	pgi(1)	phoE(1)	rpoB(1)	tonB(4)
WCHKP7E2	11	gapA(3)	infB(3)	mdh(1)	pgi(1)	phoE(1)	rpoB(1)	tonB(4)
WCHKP8F4	11	gapA(3)	infB(3)	mdh(1)	pgi(1)	phoE(1)	rpoB(1)	tonB(4)
XDR	29	gapA(2)	infB(3)	mdh(2)	pgi(2)	phoE(6)	rpoB(4)	tonB(4)
XH209	17	gapA(2)	infB(1)	mdh(1)	pgi(1)	phoE(4)	rpoB(4)	tonB(4)
YH43	new	gapA(177)	infB(119)	mdh(197)	pgi(104)	phoE(357)	rpoB(183)	tonB(~354)
ZYST1	1	gapA(4)	infB(4)	mdh(1)	pgi(1)	phoE(7)	rpoB(4)	tonB(10)

Table S4. (Excel) Information about the first 1000 whole genome sequences, retrieved from NCBI, used to investigate the occurrence and co-occurrence of PAP-Kp and ICEKp1.

Table S5 Information about representative strains of 1000 genome (NCBI) analysis, used for phylogenetic analysis across reference to Figure 3.

KP STRAINS	SEQ. TYPE	CAPSULE LOCUS (KL)	ICE_TYPE	LOCATION	MDR	ISOLATION (SOURCE)	YEAR	OUTBREAK
**HS11286**	ST11	KL103	Both	China	++	Sputum	2011	-
**A1674**	ST11	KL47	Both	China	++	ND	2013	ND
**CHS224**	ST258	KL107	Both	USA	++	ND	2014	+
**CHS81**	ST258	KL107	Both	USA	++	ND	2014	+
**CHS96**	ST258	KL107	Both	USA	++	ND	2014	+
**CHS158**	ST258	KL107	Both	USA	++	ND	2014	+
**CHS105**	ST258	KL107	Both	USA	++	ND	2014	+
**CHS218**	ST258	KL107	Both	USA	++	ND	2014	+
**CHS-38**	ST258	KL107	Both	USA	ND	ND	2014	+
**CHS202**	ST258	KL107	Both	USA	++	ND	2014	+
**KPNIH32**	ST258	KL107	Both	USA	++	Rectal swab	2013	ND
**UCI-38**	ST258	KL107	ICEKP2	USA	+	Urine	2013	+
**UCI-41**	ST258	KL107	ICEKP2	USA	++	Urine	2013	+
**UCI93**	ST258	KL107	ICEKP2	USA	+	Urine	2013	+
**UCI-33**	ST258	KL107	ICEKP2	USA	++	Urine	2013	+
**UCI-22**	ST258	KL107	ICEKP2	USA	-	Sputum	2013	+
**MGH-67**	ST258	KL38	ICEKP2	USA	ND	Pleural tissue	2013	+
**UHKPC17**	ST258	KL107	ICEKP2	USA	++	ND	2013	+
**MGH-31**	ST258	KL107	ICEKP2	USA	ND	Pleural tissue	2013	+
**UCI-19**	ST512	KL107	ICEKP2	USA	++	Urine	2013	+
**AR_0079**	ST258	KL106	ICEKP2	USA	++	ND	2015	ND
**KP_GOE_821588**	ST11	KL64	ICEKP1	Germany	ND	Rectal swab	2014	ND
**KP_GOE_822917**	ST11	KL15	ICEKP1	Germany	ND	Skin swab	2013	ND
**12208**	ST11	KL15	ICEKP1	China	-	Sputum	2013	ND
**ST101**	ST29	KL54	ICEKP1	Africa	+	Urine	2013	ND
**KP_GOE_71070**	ST101	KL17	ICEKP1	Germany	ND	Urine	2013	ND
**KP_GOE_121641**	ST101	KL17	ICEKP1	Germany	ND	ND	2013	ND
**CHS159**	ST101	KL17	ICEKP1	USA	-	Urine	2014	ND

**KP1803**	ST158	KL62	ICEKP1	Spain	+	Urine	2014	ND
**KP36**	ST14	KL2	ICEKP1	Taiwan	+	Urine	2007	ND
**MG-72**	ST15	KL64	ICEKP1	USA	ND	Urine	2013	ND
**KP985**	ST37	KL38	None	Spain	ND	Blood	2013	ND
**CHS112**	ST15	KL112	None	USA	-	ND	2014	ND
**KP995**	ST15	KL146	None	Spain	ND	Blood	2013	ND
**NH25**	ST37	KL38	None	Thailand	++	Sputum	2016	ND
**MGH83**	ST15	KL19	None	USA	++	ND	2014	ND
**MGH8**	ST309	KL42	None	USA	-	ND	2014	ND
**MGH-78578**	ST309	KL42	None		-	Sputum	2007	ND
**TGH13**	ST38	KL52	None	Greece	ND	Human habitat	2013	ND
**AATZP**	ST147	KL64	None	USA	-	Liver abscesses	2014	ND
**KP984**	ST147	KL64	None	China	-	Sputum	2009	ND

ND (not defined), ++ (resistant to all different carbapenem drugs), + (partially resistant), - (sensitive)

Table S6. Repeat sequences identified in ICEKp2 (candidates for oriT)

Bases	1st Position	Nature*	2nd Position	P-value
38	10439	F	12789	1.17e-14
31	10500	F	12850	1.91e-10
20	5872	F	42700	8.03e-04
20	47174	F	49915	8.03e-04
19	36482	R	36482	3.21e-03
18	10478	F	12828	1.28e-02
17	8655	R	8655	5.14e-02
17	10361	F	12711	5.14e-02
17	15464	R	15464	5.14e-02
17	53770	R	53770	5.14e-02
16	8567	F	42530	2.05e-01
16	43747	F	53006	2.05e-01
16	47108	F	49844	2.05e-01
15	312	R	36930	8.22e-01
15	7948	F	30244	8.22e-01
15	12750	R	32465	8.22e-01
15	12991	R	35877	8.22e-01
15	13814	F	50627	8.22e-01
15	36587	R	36587	8.22e-01
15	50298	F	50387	8.22e-01
14	1000	F	49117	3.29e+00
14	1086	R	10111	3.29e+00
14	5825	F	8566	3.29e+00
14	5866	F	8609	3.29e+00
14	6752	R	6752	3.29e+00
14	7801	R	53845	3.29e+00
14	10346	F	12696	3.29e+00
14	10695	F	33614	3.29e+00
14	10886	F	42158	3.29e+00
14	12933	R	20962	3.29e+00
14	14648	R	14648	3.29e+00
14	14662	F	42772	3.29e+00
14	15052	F	38628	3.29e+00
14	16846	F	17413	3.29e+00
14	17851	F	41369	3.29e+00

a= match direction of repeated sequence in ICEKp2 [F (forward) = direct repeat, R (reverse) = inverted repeat.

b= calculated E-value of this repeat (show the repeats with smallest E-value)

Table S7. Putative T4SS and relaxase identified in ICEKp2.

**ORF**	**Amino acids**	**Homolog proteins**	**Possible Function**
**Position in ICEKp2**			
ORF2	429	Relaxase	Nic at *oriT*
ORF17	509	TraG	Pilus assembly
ORF19	473	ICEs protein, PFL_4711	SFU
ORF20	313	ICEs protein, PFL_4710	SFU
ORF21	131	ICEs protein, PFL_4709 family	Transposition
ORF30	924	CT[b] ATPase, PFL_4706	Energy provider
ORF31	132	CT protein	Pilus assembly
ORF32	494	TrbI-like / PFL_4705	Transposition
ORF33	281	PFL_4704/TraK	SFU
ORF34	217	ICEs protein, PFL_4703	Transposition
ORF35	123	CT region protein, TIGR03750	Transposition
ORF36	117	ICEs membrane protein, PFL_4702	Assembly
ORF37	78	ICEs protein, PFL_4701	SFU
ORF38	105	ICEs protein	SFU
ORF41	252	Membrane protein PFL_4697	Transposition
ORF43	699	Coupling factor TraD	Transposition & Energy
ORF44	170	ICEs protein, PFL_4695	SFU
ORF46	205	ICEs protein, PFL_4693 family	SFU
ORF48	189	Type IV B pilus protein	Pilus assembly
ORF50	240	ICEs protein, PFL_4669 family	SFU

Table S8. Colony counts of donors, recipients and transconjugants before and after conjugation.

	Before Conjugation		After Conjugation		
**Strain as Donor**	**Donor Cfu/ml (± SD)**	**Recipient Cfu/ml ± (SD)**	**Donor Cfu/ml ± (SD)**	**Recipient Cfu/ml ± (SD)**	**Transconjugants Cfu/ml ± (SD)**
**WT**	1.6×10^9 (± 0.8)	1.5×10^8 (± 0.1)	2.6×10^9 (± 0.5)	3.7×10^7 (± 0.3)	3.6×10^2 (± 0.1)
**ΔICEKp1**	1.7×10^9 (± 1.2)	1.7×10^8 (± 0.8)	2.6×10^9 (± 1.0)	5.2×10^7 (± 0.4)	Not detected
**ΔICEKp2**	1.5×10^9 (± 0.4)	1.6×10^8 (± 0.3)	2.4×10^9 (± 0.2)	3.5×10^7 (± 0.4)	7.9×10^1 (± 1.0)
**Δmob2**	2.5×10^9 (± 0.7)	3.1×10^8 (± 1.0)	3.4×10^9 (± 0.8)	7.5×10^7 (± 0.6)	1.4×10^2 (± 0.7)
**Δmob2+mob2**	1.4×10^9 (± 0.6)	1.6×10^8 (± 1.1)	3.2×10^9 (± 0.2)	6.8×10^7 (± 0.7)	7.3×10^2 (± 0.8)
**ΔICEKp2+mob2**	2.2×10^9 (± 0.1)	2.6×10^8 (± 0.7)	2.6×10^9 (± 0.4)	7.8×10^7 (± 1.3)	8.9×10^2 (± 0.3)
**Δmob1**	2.3×10^9 (± 0.5)	3.1×10^8 (± 0.6)	2.8×10^9 (± 0.4)	5.3×10^7 (± 0.9)	Not detected
**Δmob1+mob2**	2.2×10^9 (± 1.3)	2.8×10^8 (± 1.0)	3.1×10^9 (± 0.6)	4.7×10^7 (± 1.2)	Not detected

Table S9. Primers used in this study

Name	Sequence (5' to 3')	Features		Reference
**Primers designed for ICEKp1 and ICEKp2 screening in *K. pneumoniae* clinical isolates.**				
**Int_F**	TGTTCATTCGCTCCAGTGA	Screening	ICEKp1	This study
**Int_R**	GGGTTATGGTCGCCGGGGA			
**fyuA_F**	CCTTCCCTTCCGGTTCGT			
**fyuA_R**	GCTCTTACCCTGGTCGCC			
**VirB1_F**	ATGCTTTCCACCACAGC			
**VirB1_R**	TTATTCCTCCTCCTCACGG			
**irp1_F**	CAGAAACGTGGCTCGACAAC			
**irp1_R**	CTTCGATGACTGCCTGTTGC			
**tasn_F**	CGAGTCCAGTCAGAGGAGCCA			
**Int_R**	GGGTTATGGTCGCCGGGGA			
**44710_F**	CTCTGTTCCGCCATAAACC	Screening	ICEKp2	
**44710_R**	GTTTCTTGCCGGGTATCGG			
**Int2_F**	CCACATATTCCAGCGTTGAC			
**Int2_R**	GTGAACATCAGGTGCCGATT			
**DBP2_F**	TTAGCCCTGCGTCCTTAAC			
**DBP2_R**	GAGCGTTATCAGGCCGTAT			
**mob2_F**	GAGGCTGATCGGTGTGTTG			
**mob2_R**	GTTCCTCGGTGCGCTATGA			
**MCP_F**	ATGGTCAGCAACATCCAGC			
**MCP_R**	GAAGCAACAGATGGCCGAT			
**tRNA-phe**	CTCAGTCGGTAGAGCAGG			
**Primers designed to amplify origin of transfer from ICEKp1 to construct marker plasmid (P-oriT-1).**				
**oriT-HindIII_F**	**GGCAAGCTT**CAAATAAAATGACAGTCATCATCCT	Amlification of oriT	P-oriT-1	This study
**oriT-SalI_R**	TAA**GTCGA**CGGCATCGCCCCATCAA			
**Primers used to construct knock out mutants in *K. pneumoniae* HS11286.**				
**GM_F**	CGAATTAGCTTCAAAAGCGCTCTGA	*hph* cassette: plasmid (pJTAG-Hyg) was used as template DNA	*hph*	Lab 212
**GM_R2**	AATTGGGGATCTTGAAGTTCCT			
**ICEKp1_UF**	TTTGCATTTCCTCGGCTGTC	Used to amplify upstream flank of ICEKp1	ΔICEKP1	This study
**ICEKp1_UR**	TCAGAGCGCTTTTGAAGCTAATTCGCGATGCGTAGGGTAAAATC			
**ICEKp1_DF**	AGGAACTTCAAGATCCCCAATTTCTGCCGGAAGGCGAATG	Used to amplify downstream flank of ICEKp1		
**ICEKp1_DR**	GTATGGCTGTCGATGAAGCT			

**ICEKp1-RF_F**	GACGTTATTGACCGCCAGC	Screening primer to detect ICEKp1 deletion		
**ICEKp1-RF_R**	CTGCTTATCCCTTTCGCCG			
**ICEKp2-_UF**	TTGCCGTAGTGAATATGCTG	Used to amplify upstream flank of ICEKP2	ΔICEKp2	
**ICEKp2-_UR**	TCAGAGCGCTTTTGAAGCTA ATTCGAGATGGTGAAAAATG GCGC			
**ICEKp2-_DF**	AGGAACTTCAAGATCCCCAAT TCCGGCACCACTATTTAAAG	Used to amplify downstream flank of ICEKP2		
**ICEKp2-_DR**	GTACCCTCGCAATGGACATT			
**mob2_UF**	TTTGCATTTCCTCGGCTGTC	To amplify upstream region to *mob2*	Δ*mob2*	
**mob2_UR**	TCAGAGCGCTTTTGAAGCTA ATTCGGAAGAATGAAACGTT ACCAGA			
**mob2_DF**	AGGAACTTCAAGATCCCCAAT ATCGCTCACCTGGTTGCC	To amplify downstream flank of *mob2*		
**mob2_DR**	GTTATTGCCGATTTGGGCG			
**mob2-RF_F**	CAACACTCTACCGTCACCA	To confirm precise excision of *mob2*		
**mob2-RF_R**	AGCTGGGTAGAGCCGAAGA			
**mob1_UF**	CCAGCGAGCAATAAGGCAC	For upstream flank of *mobB*	Δ*mobB*	
**mob1_UR**	TCAGAGCGCTTTTGAAGCTA ATTCGTAAAATCATGATACCT CCCTCG			
**mob1_DF**	AGGAACTTCAAGATCCCCAAT TGATTTTTAAGGAGTGGTTAT GCTG	For downstream flank of *mobB*		
**mob1_DR**	CGCTCGGTTTCAATGGCAAT			
**mob1-RF_F**	GGCTTGGTCAGTACGGATTG	For confirmation of the precise deletion		
**mob1-RF_R**	GGTGGCTGGCGATAATCGA			
**ICEKP2-_UF**	TTGCCGTAGTGAATATGCTG	Upstream flank of Int2a	Δ*int2a*	
**ICEKP2-_UR**	TCAGAGCGCTTTTGAAGCTA ATTCGAGATGGTGAAAAATG GCGC			
**Int2a_DF**	AGGAACTTCAAGATCCCCAAT TCATCCATAGTCAATTTCGTC	Downstream flank of Int2a/		
**Int2a_DR**	CTTCTGAACCGGCATTGTG			
**ICEKp2--RF_F**	GATGTCAGCGAAAGGCAAG	To confirm precise excision		
**44710_R**	GTTTCTTGCCGGGTATCGG			

**Int2b_UF**	ATACAAAGGACGCGGAAGAC	Upstream flank of int2b	*Δint2b*	
**Int2b _UR**	TCAGAGCGCTTTTGAAGCTA ATTCGCATGACGATTAGCCTT TTATAAAAT			
**Int2b _DF**	AGGAACTTCAAGATCCCCAAT TTAGACATCAAGACCTCTGGT	Downstream flank of int2b		
**Int2b _DR**	CAACATCCTCTACGCTCTGA			
**Int2b-RF_F**	CGGCAATGGAAGGAAAGAA C	Screening primers to confirm the deletion		
**Int2b-RF_R**	GCTACCCGCGATTAAGGCA			
**Primers used for the detection of integration/excision activity of ICEKp2**				
**B1**	TGAATATGCTGCCTGCGCT	Excision assay of ICEKp2	***attL***	This study
**1**	GTGAACATCAGGTGCCGATT			
**I1**[+]	CTGCACGGGACCAACATCT			
**I2**	TCGCCGTCTATCAGCAAAG		***attR***	
**B2**	GATGGGCATTTCTGAAGCC			
**Primers designed to amplify various regions of ICEKp2 for the construction of ICEKp2 marker plasmids to investigate the presence of origin of transfer. Infusion cloning method was used to construct ICEKp2 marker plasmid.**				
**PU_F**	ACTGGCCTCAGGCATTTGA	For pACYC184 backbone	Cloning	This study
**PU_R**	GTGCCTGACTGCGTTAGC			
**ICEKp2-A_F**	**ACGCAGTCAGGCAC**GGCGA AATCAGATGTGAGG	For amplifications of fragments of ICEKP2. Red colour indicates the complementary sequence of primers used to amplify pACYC184		
**ICEKp2-A_R**	**ATGCCTGAGGCCAGT**CTGCC CGAAAGGAACCACT			
**ICEKp2-B_F**	ACGCAGTCAGGCACATGTAA TAACGGTTCACTGGC			
**ICEKp2-B_R**	ATGCCTGAGGCCAGTATGAA AGCCAGTGCTATGAGT			
**ICEKp2-C_F**	ACGCAGTCAGGCACTCACTCC TCAATGTTTCCTAATA			
**ICEKp2-C_R**	ATGCCTGAGGCCAGTATGAG TGCCGACAGCTATC			
**ICEKp2-D_F**	ACGCAGTCAGGCACCGCTGC GTTCCTTATTGAAC			
**ICEKp2-D_R**	ATGCCTGAGGCCAGTTCGAG TGCCGATACATTGAT			
**ICEKp2-E_F**	ACGCAGTCAGGCACAAGAAG CGTCATCATAACCTGA			
**ICEKp2-E_R**	ATGCCTGAGGCCAGTCCTGC AACCCATGATAGCC			
**ICEKp2-F_F**	ACGCAGTCAGGCACGCATCC CGTACCTGAAGC			
**ICEKp2-F_R**	ATGCCTGAGGCCAGTCGATG ATGCAGGTGAAGTCA			

**ICEKp2-G_F**	ACGCAGTCAGGCACTTGTCTC GATCCAGTTCGAC			
**ICEKp2-G_R**	ATGCCTGAGGCCAGTCTACA CATGGCGCGAACAC			
**ICEKp2-H_F**	ACGCAGTCAGGCACTGCGTA ATAAATGTGCATCGC			
**ICEKp2-H_R**	ATGCCTGAGGCCAGTGCATA GCCAGTCGGTAGAT			
**ICEKp2-I_F**	ACGCAGTCAGGCACGACAAA CAGCGCCAGACG			
**ICEKp2-I_R**	ATGCCTGAGGCCAGTTGACT GAGGTTCTCTTGTTGT			
**ICEKp2-J_F**	ACGCAGTCAGGCACTTAGCA GCGAAGGACTTACC			
**ICEKp2-J_R**	ATGCCTGAGGCCAGTGCGAA TTTATCTGACCAGACA			
**ICEKp2-K_F**	ACGCAGTCAGGCACGCACTC GTTACCTTTATCGCA			
**ICEKp2-K_R**	ATGCCTGAGGCCAGTCAACA GGTGACATTATGCAAG			
**Primers used to amplify mob2 for the construction of complementation plasmid.**				This study
**mob2_HindIII-F**	**GGCAAGCTT**TCATTCTTCCTC TCCTTCTG	To amplify mob-2	Cloning	
**mob2_NotI-R**	ATAGTTTAGCGGCCGCGTGA GCGATCGCTATGTG			
**Primers used for point mutations in *mob2*.**				
**MOBKE-F**	GTGAGCGATCGCTATGTGAT TG	Used to amplify SOE fragment of disrupted *mob*-2	Cloning	
**MOBK^{199}-R**	CAGGCGCGTTGCCCCTAC	Substitute lysine (199) with alanine		
**MOBK^{199}-F**	GTAGGGGCAACGCGCCTG	Substitute lysine (199) with alanine		
**MOBE^{507}-F**	CCACTGTGACGCATTCAGTG AA	Substitute glutamic acid (507) with alanine		
**MOBE^{507}-R**	TTCACTGAATGCGTCACAGTG G	Substitute glutamic acid (507) with alanine		
**MOBKE-R**	TCATTCTTCCTCTCCTTCTGCC	Used to amplify SOE fragment of disrupted *mob*-2		
**Primers used for plasmid construction of complementation experiment of point disrupted mob-2**				
**PW_F**	GGTCGACGGTATCGATAAGC	To amplify the RFTool-1 plasmid	Cloning; Red colour indicates the complementary sequence of primers used to amplify RFTool-1	
**PW_R**	CGGCCGCTCTAGAACTAGT			
**MOBPW-F**	**GTTCTAGAGCGGCCG**GTGAG CGA TCGCTATGTGATTG	HD infusion cloning of *mob*^{K199E507} into RFTool-1		
**MOBPW-R**	**TCGATACCGTCGACC**TCATTC TTCC TCTCCTTCTGCC			
**Primers used to confirm various plasmids and bacterial strains.**				

**EBGNHe-F**	CCCGCTAGCGAAAAGATGTT TCGTGAAGC	pKOBEG-Apra	Plasmids	Lab 212
**EBGh3-R**	GGGAAGCTTATTATCGTGAG GATGCGTCA			
**p184_F**	ATCAGGCGGGCAAGAATGTG	pACYC184		This study
**p184_R**	GTAAGTTGGCAGCATCACCC			
**M13-F**	TGTAAAACGACGGCCAGT	Primers outside the multiple cloning site of pWSK29		Universal
**M13 -R**	CAGGAAACAGCTATGACC			
**SacB – F**	TAACAGCAGCGTGACAAGTG TAGGCCCGTAGTCTGCAAAT	Primers used to detect sacB on pflp2-Apra		
**SacB – R**	GCCCTATGGGATTCACCTTT			
**EglmS-down**	GATGACGGTTTGTCACATGG	Specific to *E. coli* genome	Bacterial	Lab 212
**EglmS-down**	TTGTATGTCTTCGCCGATCAG			

Supplementary Figures

Figure S1 Occurrence of ICEKp1 and ICEKp2 in unsequenced of *K. pneumoniae* isolated in Leicester, UK.

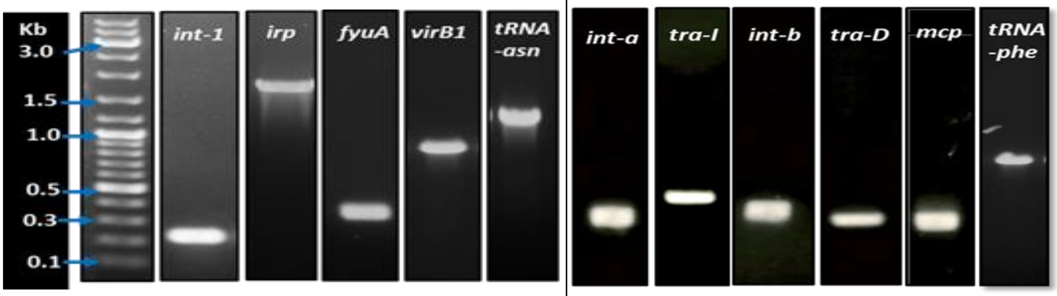

Clinical Isolates			ICEKp1					ICEKp2					
			int (0.24-kb)	*irp1* (1.9-kb)	*fyuA* (0.35-kb)	*virB4* (0.7-kb)	^{a}*tRNA*^{asn} (*1.1-kb*)	*Int2a* (0.33-kb)	*tra-I* (0.4-kb)	*Int2b* (0.35-kb)	*mob2* (0.34-kb)	*mcp* (0.3-kb)	^{c}*tRNA*^{phe} (0.67-kb)
HS11286		Control	+	+	+	+	+	+	+	+	+	+	+
MGH 78578			-	-	-	-	-	-	-	-	-	-	-
KR2170		Isolated from blood culture	+	+	+	+	+	-	-	-	-	-	-
KR2171			-	-	-	-	-	-	-	-	-	-	-
KR2172			-	-	-	-	-	-	-	+	+		
KR2173			-	-	-	-	-	-	-	-	-	-	-
KR2174			+	+	+	+	+	-	-	+[b]	+[b]	-	-
KR2175			-	-	-	-	-	-	-	-	-	-	-
KR2176			-	-	-	-	-	-	-	-	-	-	-
KR2177			+	+	+	-	+	-	-	-	-	-	-
KR2178			-	-	-	-	-	-	-	-	-	-	-
KR2179			-	-	-	-	-	-	-	-	-	-	-
KR2180			-	-	-	-	-	-	-	-	-	-	-
KR2181			+	+	+	+	+	-	-	-	-	-	-
KR2201			+	+	+	+	+	-	-	-	-	-	-
KR2202			+	+	+	+	+	-	-	-	-	-	-
KR2203			+	+	+	+	+	-	-	-	-	-	-
KR2204			-	-	-	-	-	-	+	+	+	-	+
KR2205			-	-	-	-	-	-	-	-	-	-	-
KR2206			-	-	-	-	-	-	-	-	-	-	-
KR2207			-	-	-	-	-	+	+	+	+	+	+
KR3152			-	-	-	-	-	-	-	-	-	-	-
KR3153			+	+	+	+	+	-	-	-	-	-	-
KR3154			+	+	+	+	+	-	-	-	-	-	-
KR3155			-	-	-	-	-	-	-	-	-	-	-
KR3156			-	-	-	-	-	+	+	+	+	+	+
KR3157			-	-	-	-	-	-	-	-	-	-	-
KR3158			-	-	-	-	-	-	-	-	-	-	-

**KR3159**			-	-	-	-	-	-	-	-	-	-	-
**KR3160**			+	-	-	-	-	-	-	-	-	-	-
**KR3161**			-	-	-	-	-	-	-	-	-	-	-
**KR3162**			-	-	-	-	-	-	-	-	-	-	-
**KR3163**			-	-	-	-	-	-	-	-	-	-	-
**KR3164**			-	-	-	-	-	-	-	-	-	-	-
**KR3165**			-	-	-	-	-	-	-	-	-	-	-
**KR3166**			+	-	-	-	-	-	-	-	-	-	-
**KR3167**			-	-	-	-	-	-	-	-	-	-	-
**KR3168**			-	-	-	-	-	-	-	-	-	-	-
**KR3169**			-	-	-	-	-	-	-	-	-	-	-
**KR3170**			+	-	-	-	+	-	-	-	-	-	-
**KR3171**			-	-	-	-	-	-	-	-	-	-	-
**KR3172**			+	+	+	+	+	-	-	-	-	-	-

A.

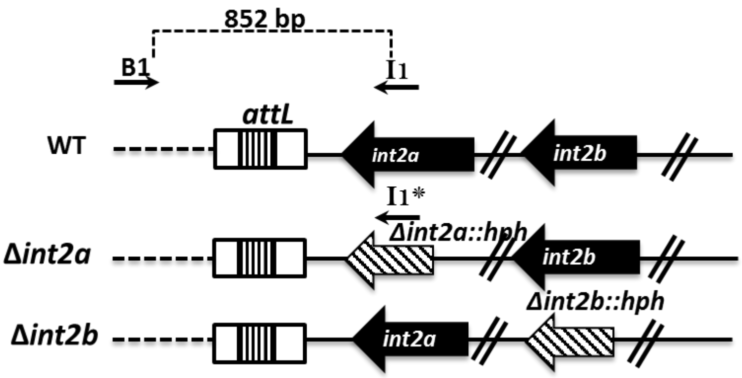

B.

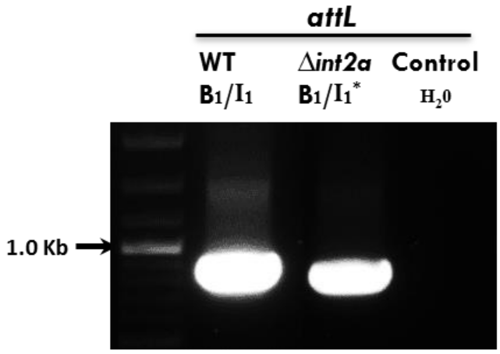

Figure S2. Gene disrupted ICEKp2 lacking *int2a* required a specific primer to amplify the attL site and this primer allowed efficient amplification of attL from chromosomal DNA of *K. pneumoniae* containing ICEKp2 Δint2a. Panel (A) shows the structure of WT and mutant ICEKp2, and the binding sites of the primers for amplification of the attL site, illustrating the need to use a primer appropriate to the strain. Panel B illustrates amplification of the *attL* site from chromosomal DNA of wild type *K. pneumoniae* (WT) and the mutant lacking int2a (Δ*int2a*) to produce amplicons of the expected sizes (852 bp and 849 bp).

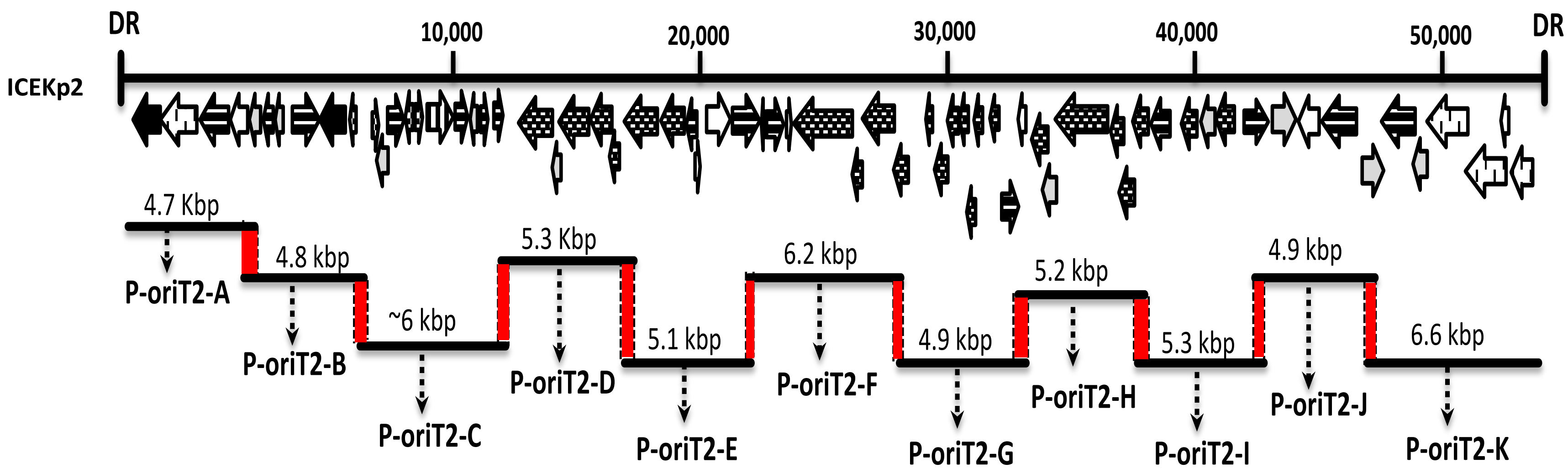

Overlapping fragments	A-B	B-C	C-D	D-E	E-F	F-G	G-H	H-I	I-J	J-K
**Size of overlapping nucleotides (bp)**	300	381	300	280	333	266	293	375	505	500

Figure S3. Cloning whole ICEKp2 into the plasmid to test presence of all possible origin of transfer in ICEKp2.
Schematics of 11 fragments from A to K with small overlapping regions (shown in red rectangles). In order to reduce the chance of truncation in *oriT*, each fragment has an overlapping region with the adjacent fragment. Sizes of overlapping regions are presented in the lower panel. These fragments were amplified using PCR (Primers are listed in Table S9) and were cloned in pACYC184 backbone using HD infusion Cloning method (Takara).

A.

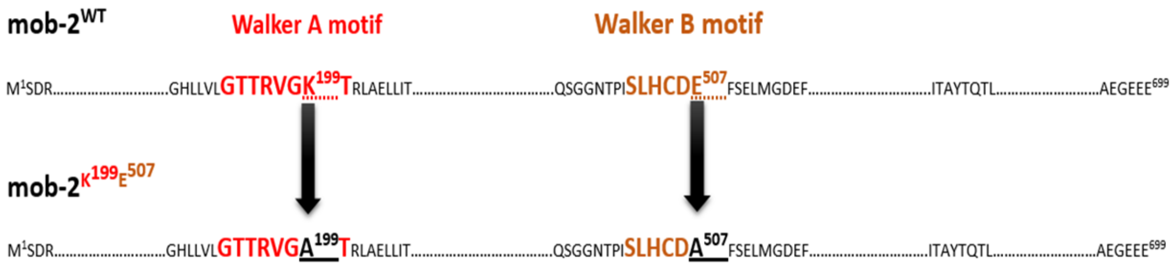

B.

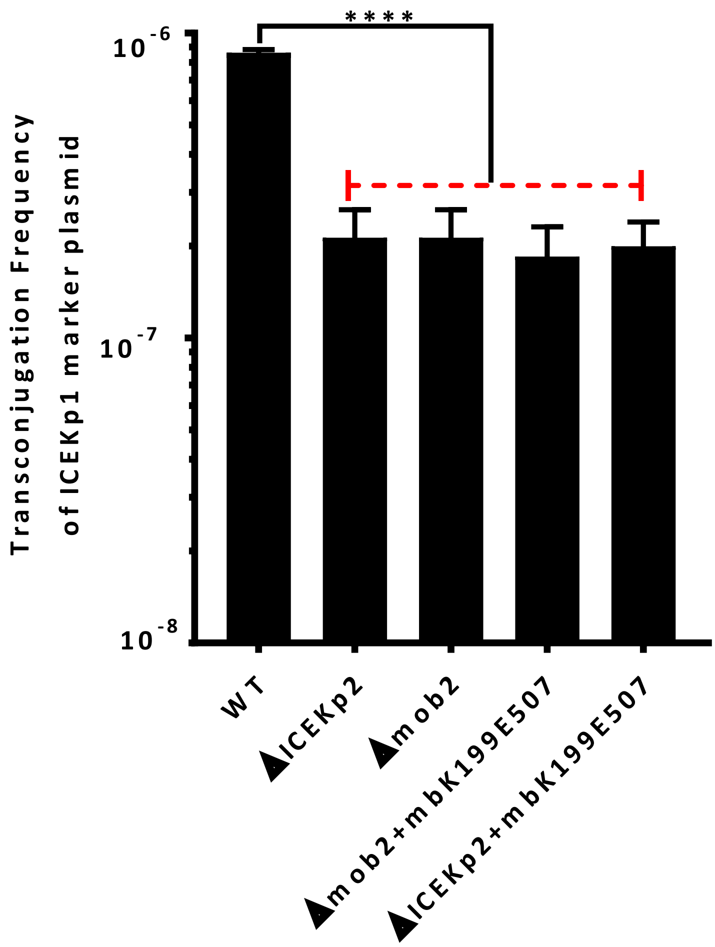

Figure S4. A variant of *mob2* with mutations in the ATP binding site (Walker A and B motifs) did not complement ICEKp1-driven plasmid transfer. A) Schematics of point mutations performed in Walker motifs of *mob2*, Lysine (K-199) and Glutamic acid (E-507) was substituted by Alanine (A199, A507). The mutated gene was cloned in RFTool-1 for complementation experiment (primers are listed in Table S9). C) Complementation of mbK199E507 into the *mob2* knock out strains did not increase ICEKp1-driven plasmid transfer.

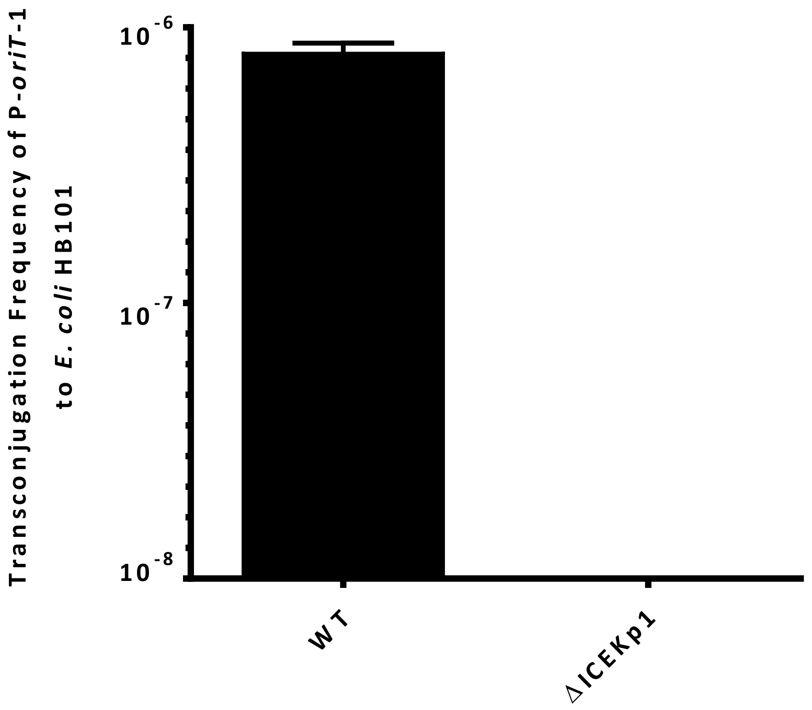

Figure S5. Validation of ICEKp1 driven conjugative mobilisation of P-oriT-1 to *E. coli*. The ability of ICEKp1 conjugation machinery to mobilise DNA was investigated by measurement of conjugal transfer of ICEKp1 marker plasmid (p-oriT-1) to *E. coli* using WT and ΔICEKp1 strains. Deletion of ICEKp1 abolished the conjugal transfer of the plasmid, demonstrating that ICEKp1 conjugation module has functionally active conjugation module. Error bar is representing the standard deviation of three independent experiments with triplicate on each occasion.
